# Modification Strategies of Carbon‐Based Electrodes From Structural Regulation to Multifunctional Integration

**DOI:** 10.1002/advs.202518189

**Published:** 2026-02-03

**Authors:** Yunlei Wang, Shitao Dou, Yifan Wu, Mingguang Wang, Taibin Wu

**Affiliations:** ^1^ School of Materials Science and Engineering Chongqing University of Arts and Sciences Chongqing China; ^2^ School of Materials Science and Engineering Chongqing University Chongqing China; ^3^ Institute for Advanced Materials and Technology University of Science and Technology Beijing Beijing China; ^4^ Department of Mechanical and Materials Engineering University of Western Ontario London Ontario Canada

**Keywords:** applications, carbon‐based electrodes, modification strategies, structural regulation

## Abstract

Carbon‐based electrodes have garnered significant attention in the field of energy storage and conversion due to their excellent electrical conductivity, chemical stability, and tunable structural characteristics. This article summarizes the modification strategies of carbon‐based electrodes, starting with structural regulation. It explores the impact of microstructural design, such as element doping, surface functionalization, structural optimization, and design of carbon‐based composite electrodes on electrode performance. Additionally, it focuses on multifunctional integration, discussing how to integrate multiple functions, including electrical conductivity, electrochemical activity, mechanical stability, and fast charge/discharge capability, into a single carbon‐based electrode system. By employing material compositing, surface modification, and nanostructural design, performance breakthroughs of carbon‐based electrodes have been achieved in the fields of lithium‐ion batteries, supercapacitors, and electrocatalysis. Finally, it summarizes the challenges and opportunities of current modification strategies and provides an outlook for future development directions, offering theoretical support and practical guidance for the high‐performance optimization of carbon‐based electrodes.

## Graphical abstract

Tracing the evolution from structural regulation to multifunctional integration, this paper systematically analyzes modification strategies for carbon‐based electrodes. It evaluates how element doping, surface functionalization, and composite material design affect the electrode performance, and offers perspectives on future applications and challenges in energy storage, electrocatalysis, and other potential areas.

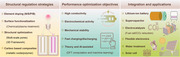

## 1 Introduction

The rapid development of renewable energy technologies and the increasing demand for sustainable energy storage and conversion systems have driven extensive research into advanced electrode materials [1, 2]. Carbon‐based electrode materials, including graphene [3], carbon nanotubes (CNTs) [4], carbon fibers (CFs) [5], and activated carbons (ACs) [6], have garnered significant attention due to their remarkable properties such as high electrical conductivity, large specific surface area, chemical stability, and structural tunability [7, 8, 9, 10]. These features make them ideal candidates for a wide range of applications, including batteries, supercapacitors, and fuel cells. However, the inherent limitations of carbon materials, such as limited active sites [11], moderate intrinsic conductivity [12], and insufficient wettability [13], pose some challenges to achieving optimal electrochemical performance.

To overcome these limitations, various modification strategies have been developed to enhance the structural and functional properties of carbon‐based materials. These strategies can be broadly categorized into chemical, physical, and structural modifications. Chemical approaches [14], such as heteroatom doping, alter the electronic structure of carbon, introducing active sites to improve reaction kinetics. Physical modifications [15], including surface functionalization and plasma treatment, enhance the interaction between the electrode and electrolyte, improving ion diffusion and electrochemical stability. Additionally, structural optimization [16], such as hierarchical pore engineering and 3D framework construction [17, 18], has proven effective in facilitating electron transport and ion mobility.

Modified carbon‐based materials have demonstrated significant advancements in energy storage and conversion applications. For instance, N‐doped graphene shows enhanced oxygen reduction reaction performance in fuel cells [19], while CNT‐metal oxide composites exhibit superior capacitance in supercapacitors [20]. Furthermore, carbon‐based materials play a crucial role in emerging technologies, such as aluminum‐ion and sodium‐ion batteries [21], offering sustainable alternatives to conventional lithium‐ion systems [22].

Recent studies have demonstrated a deep integration with carbon‐based electrode research, reflecting a key trend in the development of new‐energy conversion. Liang et al. [23] reported an osmosis‐driven green‐hydrogen production technology that is intimately linked to carbon‐based electrodes. Ordered mesoporous carbon used as the electrode substrate markedly enhances ion‐transport efficiency. Its 3D porous framework offers ideal pathways for electrolyte infiltration, while the superior electrical conductivity and chemical stability of carbon ensure long‐term, stable hydrogen generation. Zhou et al. [24] focused on interfacially super‐assembled ordered mesoporous carbon–silica hybrid membranes; through precise nanostructural design, these carbon‐based materials achieve intelligent regulation of ion transport, and their dual temperature–pH responsive behaviour provides a new concept for self‐adaptive energy‐conversion systems. The porous carbon skeleton not only improves ion selectivity but also reinforces the mechanical strength and thermal stability of the membranes. Fan et al. [25] further extended the application scope of carbon‐based electrodes in energy storage; leveraging recent advances in biomass‐derived carbons, such electrodes possess abundant active sites and tunable surface chemistry. Notably, in all these studies, carbon‐based electrodes exhibit excellent electrochemical activity and stability, playing pivotal roles in osmotic‐energy conversion, ion‐selective transport, and energy storage. Structurally, the hierarchical porosity of carbon facilitates rapid ion migration, surface functional groups enhance ion selectivity, and the conductive network guarantees efficient charge transfer. These attributes make carbon‐based electrodes an ideal platform that bridges green‐hydrogen production, smart ion transport, and high‐efficiency energy storage, providing a vital materials foundation for sustainable energy technologies.

It is worth noting that, on one hand, the excellent electrical conductivity of carbon materials provides fast electron transport channels, effectively reducing electrode polarization and enhancing rate capability. On the other hand, their abundant porous structure facilitates electrolyte infiltration and ion diffusion, which is particularly critical in supercapacitors and metal‐air batteries. Moreover, introducing heteroatoms such as N, S, and P enables precise modulation of surface charge distribution and electronic structure in carbon materials, thereby improving electrocatalytic activity and electrolyte affinity. Taking nitrogen doping as an example, pyridinic and graphitic nitrogen are widely recognized to significantly promote key reactions like the oxygen reduction reaction (ORR) and oxygen evolution reaction (OER), making them highly favored in metal‐air batteries and CO_2_ electroreduction catalysis [26].

In recent years, the application of carbon‐based electrodes in CO_2_ reduction reaction (CO_2_RR) has gained increasing attention. Against the backdrop of global climate change and carbon neutrality goals, electrocatalytic CO_2_ reduction is regarded as a green technology to convert greenhouse gases into high‐value chemicals or fuels [27]. In such systems, carbon materials serve as ideal substrates for loading metal or single‐atom catalysts, offering efficient charge transfer pathways and robust structural support. Simultaneously, constructing defective or edge‐rich carbon structures can directly activate CO_2_ reduction without metal catalysts, promising cost reduction and enhanced selectivity. Despite these advancements, challenges remain in the uniformity of modification processes, scalability for industrial applications, and long‐term stability of modified materials under operational conditions. Addressing these issues requires innovative strategies, such as green synthesis methods [28], multifunctional material designs [29], and advanced characterization techniques to correlate structure‐property relationships.

**FIGURE 1:**
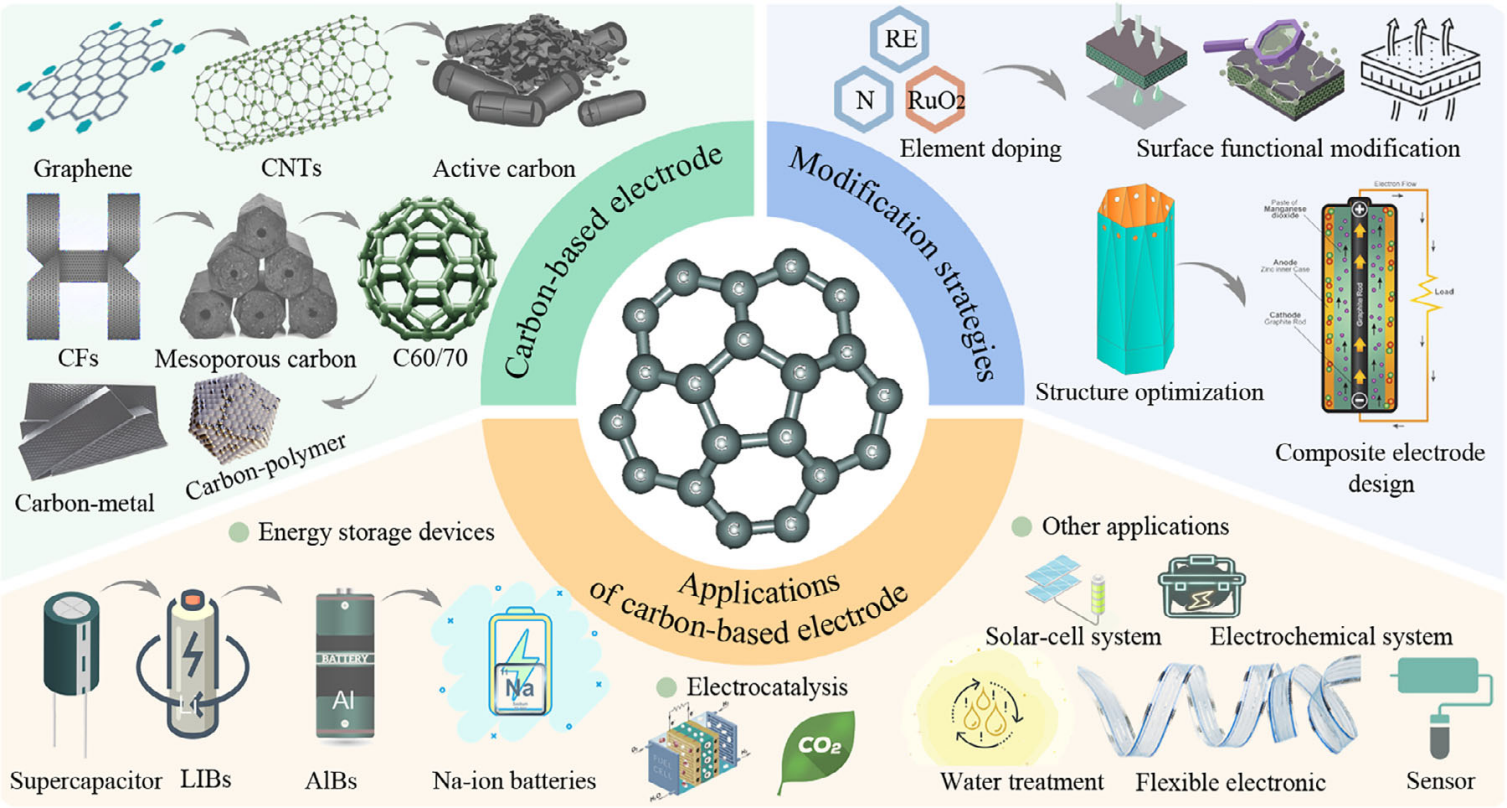
The framework of this study focuses on carbon‐based electrodes.

Based on the understanding of the current research status and development background mentioned above. This review aims to provide a comprehensive overview of the modification strategies for carbon‐based electrode materials (Figure 1), highlighting recent progress in their applications across energy storage and conversion systems. It also discusses the challenges and future directions in this field, emphasizing the potential of carbon materials as versatile platforms for multifunctional integration. To provide a clearer understanding of the paper's framework, the structure is outlined as follows: it introduces the fundamental classification of carbon‐based electrode materials and their performance characteristics. And delves into mainstream strategies, including chemical modification, physical modification, and structural optimization. Subsequently, it summarizes recent advancements in their applications, covering supercapacitors [20], lithium‐ion batteries (LIBs) [22], aluminum batteries, and electrocatalysis. Finally, it examines current challenges and limitations while offering insights into future development directions.

## 2 Types of Carbon‐Based Electrodes

Carbon‐based electrodes are a class of electrochemical electrodes with carbon materials as the core active components, which can be categorized into five major types based on structural and performance differences:

1. Traditional carbon electrodes, represented by natural graphite [30], artificial graphite [31], and amorphous carbon (e.g., anthracite coke) [32], exhibit stable conductivity and low cost. They are widely used in steelmaking arc furnaces, aluminum electrolysis cells, and fundamental electrochemical devices.
2. Porous carbon electrodes [33], including activated carbon, mesoporous carbon (e.g., CMK‐3), and ordered mesoporous carbon (OMC), leverage their high specific surface area (500–3000 m^2^/g) and tunable pore structures. These materials serve as preferred choices for supercapacitors, water treatment, and gas adsorption, offering short ion diffusion paths and excellent rate performance.
3. Nanocarbon electrodes, exemplified by graphene, CNTs, and carbon aerogels, combine ultrahigh conductivity (10^4^–10^6^ S/m), mechanical strength, and chemical stability. They demonstrate unique advantages in flexible energy storage devices, sensors, and catalytic supports.
4. Carbon‐based composite electrodes, formed by integrating carbon with metal oxides (e.g., MnO_2_/RGO) or conductive polymers (e.g., polyaniline/carbon cloth) [34], achieve synergistic effects. These composites significantly enhance specific capacity, cycling stability, and rate capability, emerging as key materials for high‐energy‐density devices.
5. Special carbon electrodes [35], such as carbon dots (CDs), carbon cloth/felt, and carbon paper, capitalize on quantum confinement effects, high porosity, or lightweight properties. They open new applications in biosensing, microbial fuel cells, and flexible electronics.

Substantially, carbon‐based electrodes, through structural engineering and composite strategies, balance conductivity, specific surface area, and chemical activity. They have become a cornerstone material system in electrochemical energy storage, catalysis, and sensing, driving continuous innovation in energy and electronic technologies.

### 2.1 Graphene and Its Derivatives

Graphene and its derivatives, due to their unique physical, chemical, and biological properties, have demonstrated extensive application potential in various fields. As is well known, graphene is a 2D material composed of a single layer of carbon atoms arranged in a honeycomb lattice, exhibiting extremely high electrical conductivity, thermal conductivity, mechanical strength, and a large specific surface area. Its main derivatives include graphene oxide (GO) and reduced graphene oxide (rGO) [34, 36]. These derivatives, through chemical modification that introduces oxygen‐containing functional groups or removes some of these groups, alter their surface activity, hydrophilicity, and electrical properties.

In the biomedical field, graphene and its derivatives have been extensively studied for applications in drug delivery, antibacterial activity, tissue engineering, and bioimaging. Additionally, Graphene and its derivatives now serve as precision tuners for carbon‐based electrodes. rGO delivers a 10^4^ S/cm conductive highway [36], only 0.5 wt% raises the electron mobility of hard‐carbon anodes by one order of magnitude and halves polarization. Oxygen‐rich GO provides proton‐coupling sites that selectively adsorb/desorb reaction intermediates, boosting CO Faradaic efficiency in CO_2_ electro‐reduction from 62% to 90%. Solution‐processable GO enables gradient porosity: electrophoretic deposition followed by in situ thermal reduction creates 20 nm–2 µm continuous channels, increasing ion‐diffusion coefficients by 2.3‐fold and power density by 45%. Flexible, ultra‐thin graphene films act as self‐supporting current collectors, eliminating metal foils and cutting total cell mass by 12% while retaining > 95% capacity after 10 000 cycles. Finally, N/S co‐doped graphene balances sp^2^ domains with defects to anchor single‐metal atoms (e.g., Fe‐N_4_), achieving 3.7 A/mg single‐site activity and offering a noble‐metal‐free route to high‐performance electrodes.

In the realm of sensors [37], graphene and its derivatives, due to their enhanced electrical properties and gas adsorption capabilities, have been employed in the development of high‐performance resistive gas sensors. These sensors are capable of rapidly responding to and detecting low concentrations of harmful gases, holding significant prospects for environmental monitoring and health protection applications.

Thanks to their exceptional electrical conductivity, mechanical flexibility, and large specific surface area, graphene and its derivatives have already yielded lab‐scale prototypes of self‐powered textiles [3], ultrasensitive e‐skins, multi‐parameter physiological patches, and flexible touch interfaces for wearable electronics, and the portfolio is expanding into energy, environmental, and biomedical arenas. While the flexible supercapacitors, VOC breath sensors, and drug‐loaded tumor patches all demonstrate clear performance gains. Yet biocompatibility has become the invisible gatekeeper to commercialization. A recent human inhalation study found no acute toxicity, giving a green light for respiratory exposure, but data are still missing on risks from chronic low‐dose contact, residual metallic impurities, and potential endocrine disruption; the newly released ISO graphene standard has only just appended bio‐testing protocols, while EU REACH registration further raises costs. Consequently, manufacturers are shifting toward graphene‐polymer masterbatch encapsulation that suppresses particle release and survives a million bending cycles, a compromise brands now accept. Over the next three years, whoever can deliver graphene composites that combine high performance [35, 36], low cost, and full ISO 10993 biocompatibility certification will turn laboratory highlights into everyday commodities.

Despite the numerous advantages of graphene and its derivatives [38, 39], their biosafety and cytotoxicity still require further investigation. For example, the aqueous dispersibility and potential cytotoxicity of graphene are key issues that need to be addressed in its biomedical applications. Future research should focus on optimizing the preparation methods of these materials, improving their biocompatibility, and exploring their multifunctional integration in different fields.

**FIGURE 2:**
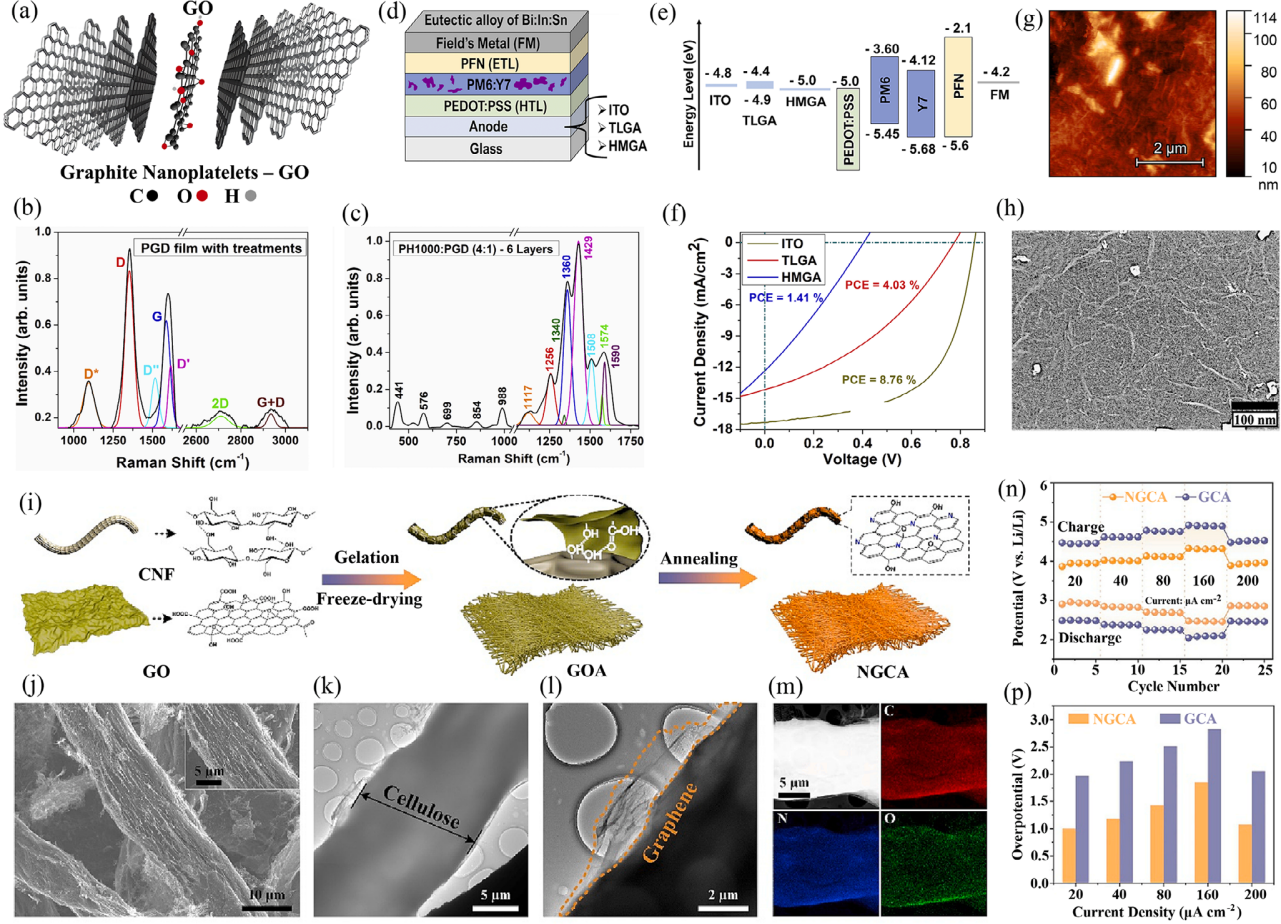
(a) Chemical structure of PGD. (b, c) Raman spectra of PGD (HI and UV‐ozone plasma treatments) and HMGA. (d) Device architecture. (e) Energy level diagram. (f) OSCs J‐V curves concept test for ITO, TLGA, and HMGA. (g) The 5 × 5 µm AFM morphology of PGD film. (h) FESEM morphology of treated PGD film. Reproduced with permission [40]. Copyright 2025, Elsevier. (i) The preparation process of NGCA. Microstructure and chemical composition of NGCA and GCA of (j) SEM image, (k, l) TEM images. (m) EDS element mapping. (n) Comparison of rate features at varying current densities. (p) Overpotential comparison at varying current densities. Reproduced with permission [42]. Copyright 2023, Elsevier.

Recently, Guerra‐Him et al. [40] introduced a processable graphene derivative (PGD) as an alternative to ITO electrodes in organic solar cells (OSCs) (Figure 2a). The team mechanically synthesized PGD and suspended it in water to fabricate two types of graphene anodes: a three‐layer graphene anode (TLGA) and a hybrid multilayer graphene anode (HMGA) (Figure 2d). The TLGA was composed of PGD and the conductive polymer PH1000 (Figure 2b,c), and was prepared by drop‐casting and UV‐ozone plasma treatment. The HMGA was fabricated by mixing PH1000 and PGD at a 4:1 volume ratio and depositing the mixture by spin‐coating. Both anodes exhibited good optical transmittance and low electrical resistance, with values of 74% and 170 Ω/sq for TLGA, and 79% and 134 Ω/sq for HMGA.

In OSCs based on PM6:Y7, TLGA, and HMGA were tested as alternative anodes. The results showed that the power conversion efficiencies (PCE) of TLGA and HMGA were 4.0% and 1.4%, respectively, significantly lower than the 8.7% achieved with the reference ITO anode (Figure 2e). Nevertheless, these preliminary results indicate that PGD has potential as an ITO alternative, particularly in terms of cost reduction and flexibility. The study also highlighted the need to further optimize the synthesis process of PGD, as well as the fabrication processes of the electrodes and OSCs (Figure 2f), to enhance their performance in photovoltaic devices [41]. In addition, atomic force microscope (AFM) imaging of the treated PGD film revealed a relatively high roughness of ∼10 nm. Subsequent deposition of the PH1000 layer reduced the overall roughness to ∼6 nm, yielding a more uniform electrode morphology that ensures better contact with adjacent device layers while simultaneously enhancing the electrical conductivity of the TLGA. (Figure 2g). The surface morphology of different electrode materials was also observed, and these morphological features may affect charge transport and device stability (Figure 2h).

In another representative study, Yu et al. [42] reported a novel 3D self‐supported graphene carbon aerogel (NGCA) for enhancing the catalytic activity and cycling stability of lithium–carbon dioxide (Li─CO_2_) batteries (Figure 2i). The team synthesized NGCA via a one‐step pyrolysis method and employed it as the cathode catalyst for Li─CO_2_ batteries. Theoretical simulations and experimental analyses indicated that this NGCA can modulate the electronic structure of graphene, reducing the free energy changes of reactants/intermediates. The inherent oxygen‐containing functional groups in the graphene aerogel, in synergy with the nitrogen dopants, further stabilize CO_2_‐related intermediates and enhance catalytic activity. NGCA features a 3D hierarchical porous structure (Figure 2j‐l), which not only ensures good electrical conductivity but also provides a large specific surface area, exposing numerous active sites (Figure 2m). The Li─CO_2_ battery integrated with the NGCA cathode demonstrated significantly improved initial energy efficiency (∼78.46%) and excellent cycling stability exceeding 1500 h (at 20 µA cm^−2^) (Figure 2n, p). This study offers new insights for the development of efficient and low‐cost cathode catalysts for Li─CO_2_ batteries and elucidates the potential catalytic mechanisms of carbon‐based metal‐free catalysts. Additionally, a series of studies have demonstrated that graphene and its derivatives hold great potential in the field of optoelectronics [40, 41, 42], which is worth paying attention to, especially in enhancing the performance of OSCs. By optimizing the synthesis and processing methods of the materials, the efficiency and stability of the devices can be significantly improved.

### 2.2 Carbon Nanotubes (CNTs)

Carbon nanotubes (CNTs), as carbon‐based electrode materials, have shown great potential in energy storage and conversion devices. The unique 1D nanostructure of CNTs offers excellent electrical conductivity, high mechanical strength, large surface area, and good chemical stability, making them ideal electrode materials for applications such as LIBs [22], fuel cells [19], and supercapacitors [20].

In LIBs, CNTs can serve as anode materials, providing fast pathways for lithium‐ion diffusion and a stable structure, thereby achieving high capacity and long cycle life [22, 42]. Additionally, CNTs can be used as conductive additives for cathode materials to enhance the conductivity and overall performance of the electrodes. In supercapacitors [20], the high surface area of CNTs helps increase the contact area between the electrode and the electrolyte, improving energy density. At the same time, the excellent electrical conductivity of CNTs contributes to increasing power density, enabling supercapacitors to charge and discharge rapidly. In fuel cells, CNTs can act as catalyst supports, offering numerous active sites to enhance the efficiency of catalytic reactions. Furthermore, CNTs can also be used as bipolar plate materials to improve the conductivity and durability of fuel cells [4, 19].

Despite the many advantages of CNTs in electrode materials [43, 44, 45], their practical application still faces some challenges, such as the cost of large‐scale production, dispersion issues during electrode preparation, and environmental and health impacts. Therefore, future research needs to focus on improving the preparation efficiency of CNTs, optimizing electrode design, and exploring their potential applications in different energy devices.

**FIGURE 3:**
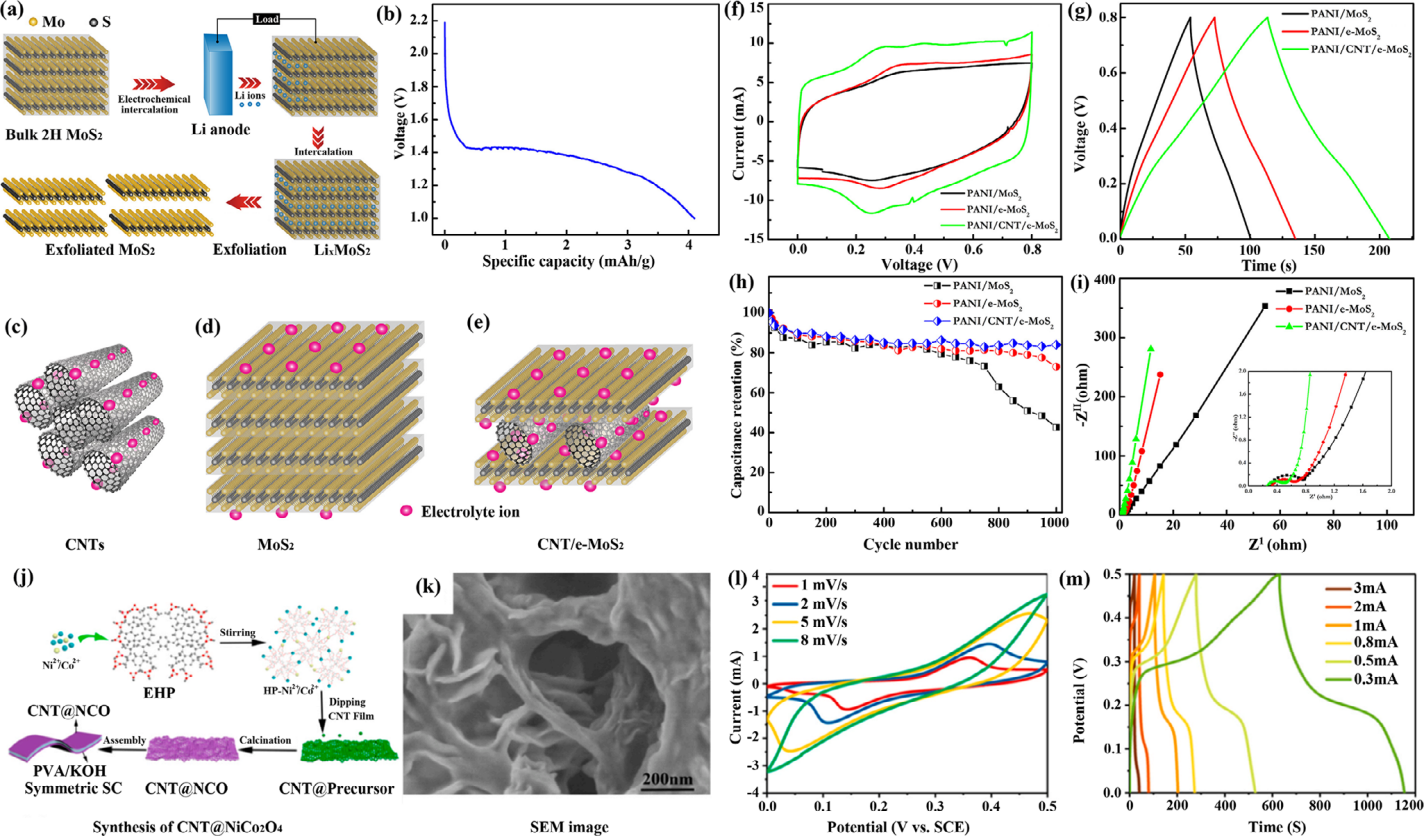
(a) Schematic representation of exfoliation of bulk MoS_2_. (b) Galvanostatic discharge curve for Li^+^ intercalation. (c–e) Comparison of CNTs, MoS_2,_ and CNT/e‐MoS_2_ composite as electrodes. (f–i) Electrochemical performance of PANI/MoS_2_, PANI/e‐MoS_2_ and PANI/CNT/e‐MoS_2_ based symmetric supercapacitors in 1 M H_2_SO_4_ electrolyte. Reproduced with permission [46]. Copyright 2022, Elsevier. (j) Synthesis and preparation of CNT@NiCo_2_O_4_‐based composite and its application for SC, (k) SEM analysis. (l, m) electrical measurements of the synthesized CNT@NiCo_2_O_4_‐based hybrid, respectively. Reproduced with permission [47]. Copyright 2020, American Chemical Society.

Jasna et al. [46] developed a novel polyaniline (PANI) wrapped CNT/exfoliated molybdenum disulfide (MoS_2_) nanosheet composite (PANI/CNT/e‐MoS_2_) for use as electrodes in high‐performance supercapacitors (Figure 3a). This composite material was prepared using a simple in situ oxidative polymerization method, and the exfoliation of single/few‐layer MoS_2_ nanosheets and the incorporation of CNTs in the PANI/e‐MoS_2_ composite were confirmed using scanning and transmission electron microscopy techniques. The electrochemical properties of the PANI/CNT/e‐MoS_2_ electrode were evaluated using cyclic voltammetry and galvanostatic charge/discharge techniques [31, 32]. The composite electrode exhibited a high specific capacitance and also demonstrated good rate capability from 1 to 10 A/g. A symmetric supercapacitor assembled using the PANI/CNT/e‐MoS_2_ composite electrode showed good cycling stability, with an 80% capacity retention after 4000 cycles, and delivered an energy density of 11.8 Wh/kg and a power density of 3785 W/kg.

In addition, the relationship between the specific capacity and current density of the electrode materials was also investigated, indicating that the material has a high specific capacity (Figure 3b). The schematic diagrams (Figure 3c) were used to illustrate the microstructure of CNTs/MoS_2_ nanosheets, elucidating how CNTs are uniformly distributed on the MoS_2_ nanosheets (Figure 3d,e). And it displays the electrochemical performance of different electrode materials, including cyclic voltammetry curves (Figure 3f,g), galvanostatic charge/discharge (GCD) curves, as well as their cycling stability and electrochemical impedance spectroscopy (EIS) analysis. These results demonstrate that the CNTs/MoS_2_ electrode material possesses excellent electrochemical properties, including high specific capacitance, good rate capability, and cycling stability [8, 9].

The high power density characteristics of the CNTs/MoS_2_ electrode material were further confirmed by the charge/discharge times at different current densities (Figure 3h). It shows the capacitance retention rate and Nyquist plots of the electrode materials at different cycle numbers (Figure 3i), respectively, further proving the stability and low internal impedance of the CNTs/MoS_2_ electrode material.

Overall, this work indicates that the studied CNTs/MoS_2_ electrode material has significant electrochemical advantages in supercapacitor applications, including high specific capacitance, good cycling stability, and low internal impedance [15, 16, 17, 18]. These characteristics make it a strong candidate for high‐performance supercapacitor electrode materials.

In the research of CNT electrodes, Hu et al. [47] synthesized the CNT@NiCo_2_O_4_ composite material via a hydrothermal method and calcination process, leveraging the electrical conductivity of CNTs and the electrochemical activity of nickel cobalt oxide to form a composite with excellent electrochemical properties (Figure 3j). The scanning electron microscope (SEM) images reveal the distribution of CNTs within the composite, indicating that CNTs form a good conductive network [19, 40], which helps to enhance the electrical conductivity of the material (Figure 3k). Moreover, the cyclic voltammetry curves show the electrochemical activity of the material at different scanning rates, demonstrating its good capacitive characteristics (Figure 3l).

The galvanostatic charge/discharge curves illustrate the capacitive performance of the material at different current densities, indicating that the material has high electrochemical stability and capacitive performance (Figure 3m) [27]. In the future, the CNT@NiCo_2_O_4_ composite with excellent performance, through optimized synthesis processes and structural design, exhibits superior electrochemical properties and is suitable for energy storage devices such as supercapacitors [20].

### 2.3 Carbon Fibers (CFs)

Carbon fiber (CF) is a high‐performance carbon‐based material [48, 49, 50], widely used as electrode material, especially in supercapacitors and fuel cells [19, 20]. CF has a high specific surface area, excellent electrical conductivity, and chemical stability, making it an ideal electrode material.

First, the high specific surface area of CF increases the contact area between the electrode and the electrolyte, thereby enhancing the charge storage capacity [51]. Second, CF has excellent electrical conductivity, which can rapidly transfer electrons, reduce resistance, and improve electrochemical performance [52]. Additionally, CF exhibits good chemical stability in various chemical environments, allowing it to maintain its performance in a wide range of applications. However, CF also has some limitations. For instance, its cost is relatively high, and in some cases, it may need to be combined with other materials to improve performance. Moreover, the preparation process of CF can be quite complex [53], requiring precise control of conditions to achieve optimal performance.

Overall, CF, as a carbon‐based electrode material, shows great potential in the field of electrochemical energy storage. With further research and development, CF is expected to play a greater role in high‐performance electrode materials.

**FIGURE 4:**
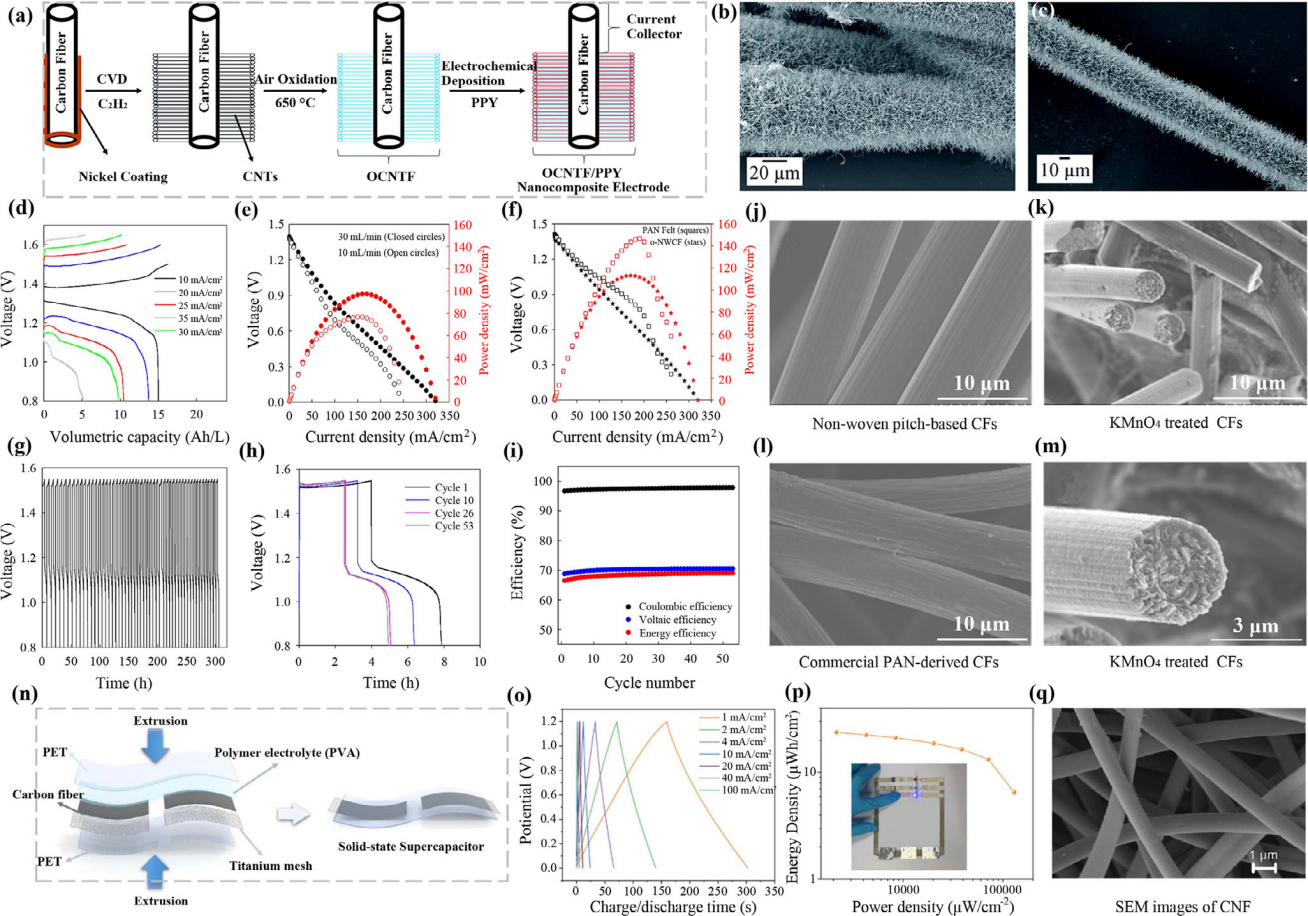
(a) Pictorial representation of the fabrication of the electrode. (b, c) SEM images of CNTF. Reproduced with permission [54]. Copyright 2016, The Royal Society of Chemistry. (d) Charge–discharge curves at 30 mL/min. (e) polarization curves at varying flowrates. (f) Polarization curves at 30 mL/min for RFBs with KMnO_4_‐treated NWCF electrodes and PAN‐derived carbon felt electrodes. (g–i) Cycling performance at 25 mA/cm^2^ and 60 mL/min. (j) SEM micrographs of non‐woven pitch‐based CFs. (k, m) KMnO_4_‐treated non‐woven pitch‐based CFs. (i) Commercial PAN‐derived carbon felt. Reproduced with permission [56]. Copyright 2025, The Royal Society of Chemistry. (n) Preparation of SC. (o) GCD curve. (P) Ragone plot with inset showing a blue LED constructed and powered by the device. (q) SEM image of a piece of CNF. Reproduced with permission [60]. Copyright 2021, American Chemical Society.

In terms of CF carbon‐based electrodes [54], it illustrates the process for fabricating OCNTF/PPY nanocomposite electrodes (Figure 4a). Initially, a layer of nickel is coated on the surface of the CF using chemical vapor deposition (CVD) technology. Then, this nickel coating serves as a catalyst for the growth of CNTs on the CF. Subsequently, these CNTs undergo air oxidation at a high temperature of 650°C, resulting in oxidized carbon nanotubes (OCNTF), and the SEM images of CNTF are shown in Figure 4b,c. Finally, polypyrrole (PPY) is deposited on the surface of OCNTF through electrochemical deposition, yielding the OCNTF/PPY nanocomposite electrode. This electrode structure is designed to enhance electrochemical performance and can act as an efficient current collector, suitable for a variety of electrochemical applications [55].

Recently, Williams et al. [56] explored a low‐cost non‐woven pitch‐based CF (NWCF) electrode material for redox flow batteries (RFBs). The research team used a scalable and cost‐effective melt‐blowing process to produce NWCF electrodes from petroleum pitch. Compared to commercial polyacrylonitrile (PAN)‐based CF felt, these pitch‐based CFs have higher graphitization content, tensile strength, and electrical conductivity, while also significantly reducing greenhouse gas emissions.

Experimental results indicate that under unoptimized conditions, RFBs using NWCF electrodes had slightly lower voltage and power density in zinc iodide electrolyte compared to those using PAN‐derived CF felt (Figure 4d–f). However, after oxidation treatment, the battery performance of NWCF electrodes in vanadium electrolyte was nearly equivalent to that of commercial PAN‐derived CF felt (Figure 4g‐i). This suggests that NWCF electrodes, due to their low‐cost precursor and more economical processing methods, offer a promising solution for reducing the cost of RFB electrode materials [57, 58]. With further optimization, these electrodes are expected to deliver improved battery performance. It is worth noting that this study presents a new type of CF electrode material that is low‐cost and environmentally friendly, demonstrating performance comparable to or even better than existing technologies in redox flow batteries, providing a new direction for the development of future energy storage technologies [59].

The microscopic structures of different types of CFs were analyzed and compared using SEM images, including untreated and KMnO_4_‐treated NWCFs (Figure 4j) and commercial PAN‐derived CFs (Figure 4l). It was observed that the diameters of both types of fibers were around 10 micrometers. However, the pitch‐based CFs exhibited a rougher and more irregular surface structure. The nonwoven pitch‐based CFs showed an increase in surface roughness (Figure 4k), which may be attributed to the introduction of more surface functional groups by the oxidation treatment. After KMnO_4_ treatment, the PAN‐derived CFs exhibited distinct granular structures on their surfaces (Figure 4m), likely due to polymer degradation or rearrangement caused by surface oxidation.

Moreover, the study revealed the differences in the microscopic structures of CFs with different types and treatments. These differences may affect the electrochemical performance of CFs, such as in redox flow batteries. In particular, KMnO_4_ treatment can alter the surface characteristics of CFs, potentially enhancing their electrochemical activity.

In addition, Li et al. [60] developed a solid‐state supercapacitor based on CF and carbon nanofibers (CNF), and investigated its fabrication process (Figure 4n), charge/discharge behavior (Figure 4o), the relationship between energy density and power density (Figure 4p), and the morphological characteristics of CNF (Figure 4q). The study revealed that with the increase of current density, the charge/discharge time is significantly shortened, indicating that the supercapacitor has a relatively fast charge/discharge rate. Moreover, as the power density increases, the energy density decreases, which is a typical characteristic of supercapacitors [20, 61]. The SEM images show that the diameter of CNF is 1 µm, with good uniformity and dispersion, which is beneficial for enhancing the electrochemical performance of the electrode material. In summary, the fabrication method, electrochemical performance, and microstructure of the electrode material of this novel solid‐state supercapacitor indicate that it has good charge/discharge performance and energy density, and is a promising energy storage device.

### 2.4 Activated Carbon (AC)

Activated carbon (AC) has become an important choice for carbon‐based electrode materials due to its high specific surface area, rich pore structure, and good physical and chemical stability, especially in the field of supercapacitors, where it has been widely studied [59, 60, 61]. In recent years, researchers have significantly improved the electrochemical performance of AC through different preparation methods and material optimizations.

Moreover, in terms of preparation methods and performance optimization, AC prepared from biomass waste (such as coconut shells, seaweed, and sycamore leaves) has shown excellent performance in supercapacitors. For instance, Manimekala et al. [61] used coconut shells as raw material and prepared AC with a specific capacity of 235 F/g at a current density of 1 A/g through KOH activation at 800°C for 60 min. Additionally, Sun et al. [62] prepared AC with a honeycomb structure from yeast cells as a precursor, achieving a specific capacity as high as 330 F/g with almost no capacity decay after 1000 cycles. Zheng et al. [63] used ZnCl_2_ pre‐activation and CO_2_/steam secondary activation methods to prepare AC from natural coconut shells, achieving a specific capacity of up to 278 F/g in 6 mol/L KOH electrolyte. This combined method not only increased the specific surface area of the AC but also optimized its pore structure, thereby enhancing its electrochemical performance. Zhang et al. [64] prepared AC with both a high specific surface area of ∼2773 m^2^/g and high conductivity of ∼912 S/m from a pitch/polyacrylonitrile (PAN) mixture precursor through a selective chemical etching strategy. The assembled symmetric supercapacitor achieved an areal capacitance of 2.8 F/cm^2^ under a high loading of 10 mg/cm^2^, with no capacity decay after 50 000 cycles.

In terms of application and performance enhancement, AC mainly functions through double‐layer energy storage mechanisms in supercapacitors. Its high specific surface area and pore structure enable it to store a large amount of charge. For example, Hou et al. [65] prepared oxygen‐rich AC through rapid KOH activation, achieving a specific capacity as high as 370 F/g at low current densities. Additionally, Wang et al. [66] synthesized AC with graphitized 3D hierarchical porous structures, achieving energy and power densities of 22.9 Wh/kg and 23 kW/kg, respectively. The combination of AC with other materials (such as graphene, CNTs, etc.) can further enhance electrode performance. For instance, in flow motor capacitive deionization desalination (FCDI) [67], the combination of AC with CNTs significantly improved charge transfer efficiency and enhanced desalination performance. Moreover, AC has also been applied in flexible solid‐state supercapacitors. For example, Pang et al. [68] used AC as the negative electrode to construct an asymmetric supercapacitor that achieved a specific capacitance of 210 mF/cm^2^ at a current density of 0.3 mA/cm^2^, demonstrating good rate performance and cycling stability.

Despite the significant progress of AC in electrode materials, it still faces some challenges. For instance, how to further improve its conductivity, optimize its pore structure to meet higher energy density requirements, and reduce preparation costs. In addition, with the development of flexible electronic devices and wearable technology, the development of high‐performance, flexible AC electrode materials will become an important research direction in the future.

### 2.5 Carbon Aerogel

Carbon aerogel, as carbon‐based materials with unique 3D porous structures, have garnered significant attention in the field of electrode materials, especially in supercapacitors, due to their high specific surface area, low density, excellent electrical conductivity, and good mechanical properties in recent years. Among them, the sol‐gel method is one of the most commonly used methods for preparing carbon aerogel [69]. By controlling the concentration of the precursor solution, the amount of crosslinking agent, and the drying, conditions the pore structure and micromorphology of the carbon aerogel can be regulated. For instance, carbon aerogels with high specific surface area and excellent electrochemical performance can be prepared from natural cellulose through the sol‐gel method and carbonization treatment [70].

The electrochemical performance of carbon gels mainly depends on their pore structure and conductivity [71]. Their 3D porous structure provides a large number of active sites, which is conducive to the transportation and adsorption of electrolyte ions, thereby increasing the specific capacitance. In addition, the high conductivity of carbon aerogels helps to facilitate rapid electron transport, further enhancing electrode performance. For example, by optimizing the preparation process [72], the specific surface area of carbon aerogels can reach more than 2000 m^2^/g, and the specific capacitance can reach more than 300 F/g. Carbon aerogels exhibit excellent electrochemical performance in supercapacitors, including high specific capacitance, good cycling stability, and excellent rate performance. Moreover, carbon aerogels have also been applied as anode materials in LIBs [73, 74], showing high specific capacity and good cycling stability. For instance, lignin‐based carbon gels exhibit a reversible capacity as high as 1109 mAh/g in LIBs [75].

To further enhance the performance of carbon aerogels, researchers have adopted various optimization strategies [72, 76]. For example, by preparing composite materials, such as combining carbon aerogels with materials like graphene and CNTs, the conductivity and mechanical properties of the electrodes can be significantly improved [77]. Additionally, by controlling the carbonization temperature and time, the pore structure of the carbon aerogels can be optimized, thereby enhancing their electrochemical performance [78].

**FIGURE 5:**
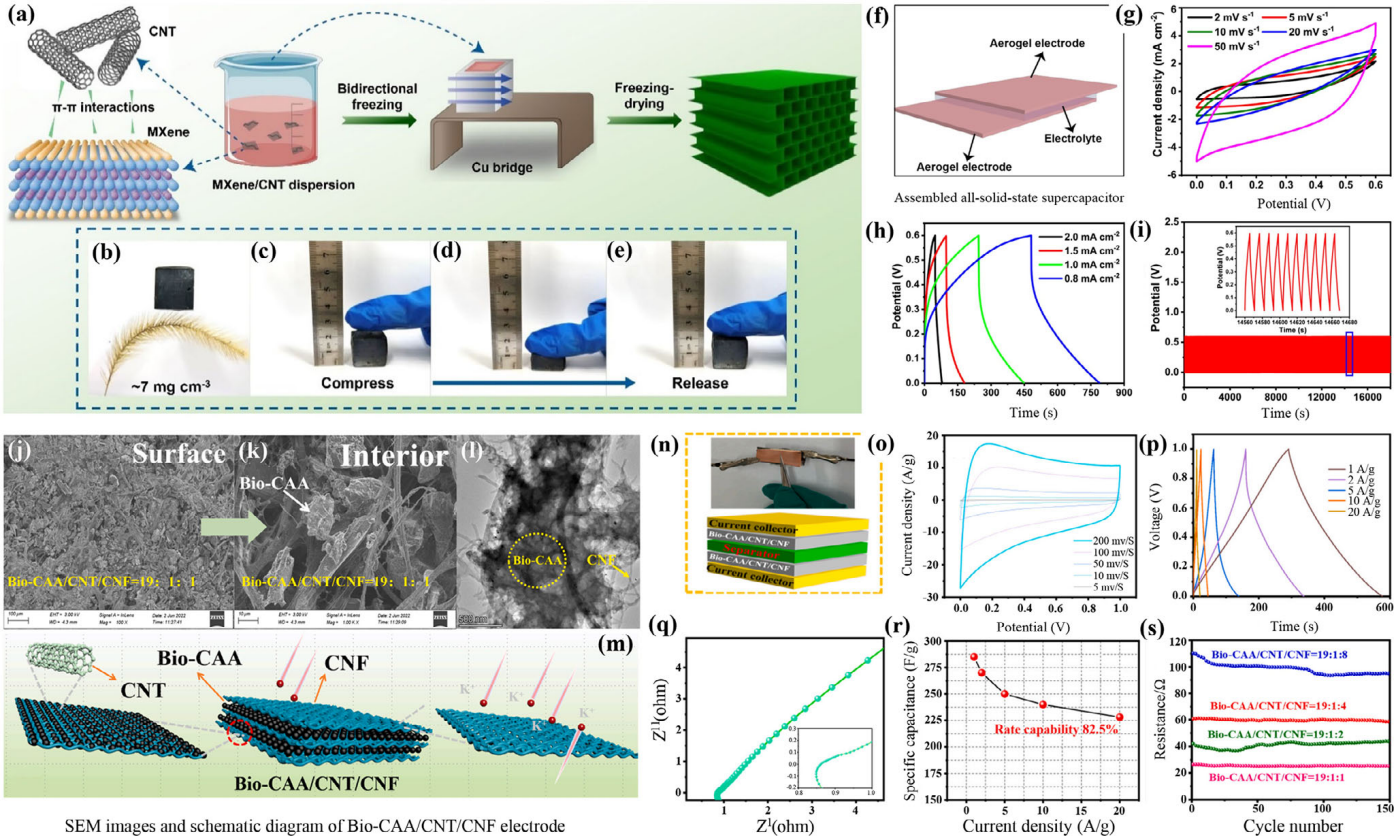
(a) The fabrication process for MXene/CNT aerogels. (b) MXene/CNT aerogel can be rested on the Setaria grass. (c–e) The photograph of the compression‐recovery process for MXene/CNT aerogel. (f) The diagram of assembled all‐solid‐state supercapacitor. (g) CV curves at scan rates of 2, 5, 10, 20, 50 mV s^−1^ of the supercapacitor. (h) GCD curves at different current densities of the supercapacitor. (i) Cycling stability of the supercapacitor. Reproduced with permission [79]. Copyright 2023, Springer. (j) SEM images of Bio‐CAA/CNT/CNF electrode of the surface. (k) The interior. (l) A low‐magnification TEM image. (m) Schematic diagram of the composite structure. (n) Schematic diagram of the SSC structure. (o) CV curves. (p) GCD curves. (q) Nyquist plot. (r) Specific capacitance. (s) Resistance stability test. Reproduced with permission [80]. Copyright 2024, Elsevier.

In a representative study, Xu et al. [79] reported a CNT/CNF composite electrode material based on CNTs and CNFs (Figure 5a). By mixing the dispersions of CNTs and CNFs and then undergoing a freeze‐casting process, a Ca‐bridged structure was formed [70, 76], and the final composite electrode material was obtained through freeze‐drying. The structural characteristics and electrochemical properties of the composite electrode were investigated. The results showed that the composite electrode material was very lightweight, with a weight of only about 7 mg/cm^2^ (Figure 5b). It also demonstrated good flexibility (Figure 5c) and compressibility (Figure 5d), and the recovery after compression indicated that the material had good elasticity (Figure 5e). A schematic diagram of the electrode material assembly was simulated (Figure 5f), and further electrochemical performance test results suggested that the composite electrode had good capacitive characteristics (Figure 5g). Galvanostatic charge/discharge curves (GCD) at different current densities indicated that the composite electrode had high electrochemical stability and capacitive performance (Figure 5h). Additionally, the relationship between capacitive density and voltage at different scanning rates further confirmed the capacitive characteristics of the composite electrode (Figure 5i).

Recently, Lv et al. [80] proposed a new method for preparing flexible supercapacitor electrode materials, which are composites synthesized from biomass carbon aerogels, CNTs, and CNFs through a one‐step self‐assembly approach [81]. SEM images of the composite electrode material revealed that it has a rich porous structure (Figure 5j), and SEM image further demonstrated the porous structure of the material (Figure 5k). Additionally, different structural levels of the electrode were labeled, including Bio‐CAA, CNF, and Bio‐CAC/CNT/CNF (Figure 5l). The study of the electrode material's Nyquist plot indicated that the material has low charge transfer resistance (Figure 5m). The relationship between current density and specific capacitance of the electrode material showed that it has a high specific capacitance at different current densities (Figure 5n). The voltage versus time relationship of the electrode material reflected its good rate performance (Figure 5o). Furthermore, its cycling stability was tested, indicating that it can maintain good capacitive performance after long‐term cycling (Figure 5p). The EIS of the electrode material reflected information about its charge transfer resistance and diffusion processes (Figure 5q), showing the change in specific capacitance with increasing current density, and at high current densities, the material maintained 82.5% of its specific capacitance [82, 83], demonstrating good rate performance (Figure 5r). Additionally, the stability of the curve can reflect the resistance changes of the material after multiple charge/discharge cycles, thereby evaluating its cycling stability (Figure 5s).

This study successfully developed a new type of flexible supercapacitor electrode material with high capacitance, good cycling stability, and excellent mechanical properties. It provides a potential solution for energy storage in flexible and wearable electronic devices. The research also demonstrates the potential of biomass materials in the preparation of high‐performance electrode materials, offering a new direction for the development of sustainable and environmentally friendly energy storage devices in the future [84].

## 3 Modification Strategies of Carbon‐Based Electrodes

The modification strategies for carbon‐based electrodes mainly involve the regulation of pore structure [85], doping with non‐metal elements [86], and composite formation with metal oxides [87]. These strategies can achieve the bifunctionalization of carbon‐based electrode materials [88], optimizing their energy storage and electrochemical performance in the fields of supercapacitors and electrocatalysis. For instance, by controlling the pore structure, the spatial capacitance charge density of porous carbon can be significantly enhanced. Doping with non‐metal elements such as N and S helps to improve the electrochemical activity and stability of the electrodes. Furthermore, the composite with materials like metal oxides can further enhance the conductivity and electrochemical performance of the electrodes [89, 90]. These modification methods are instrumental in addressing energy storage issues and improving electrochemical performance, representing an important research direction for carbon‐based electrode materials in the field of electrochemistry.

### 3.1 Element Doping

In the optimization strategies for carbon‐based electrode materials, elemental doping is an effective method that can significantly enhance the electrochemical performance of the materials. By doping with non‐metal elements such as N and S [86, 91], additional free electrons can be introduced, increasing the carrier concentration of the material and thereby improving the electronic conductivity of silicon‐based anodes [92]. For example, after nitrogen doping achieved via a one‐step process using high‐frequency thermal plasma technology, the electronic conductivity of silicon materials is enhanced, which facilitates rapid electron transfer, reduces electrode polarization, and consequently improves rate performance. Additionally, by forming a polypyrrole hydrogel through the polymerization of pyrrole and introducing phytic acid and FeCl_3_ as phosphorus and iron sources respectively [93], followed by pyrolysis of the hydrogel in nitrogen and etching with sulfuric acid, nitrogen and phosphorus dual‐coordinated iron active sites can be embedded in carbon nanosheets. This N and P co‐doping approach can accelerate the kinetics of the oxygen reduction reaction and achieve high catalytic activity [94]. These studies demonstrate that elemental doping strategies can not only improve the electrochemical activity and stability of carbon‐based electrode materials but also optimize their pore structure, further enhancing the conductivity and electrochemical performance of the electrodes.

**FIGURE 6:**
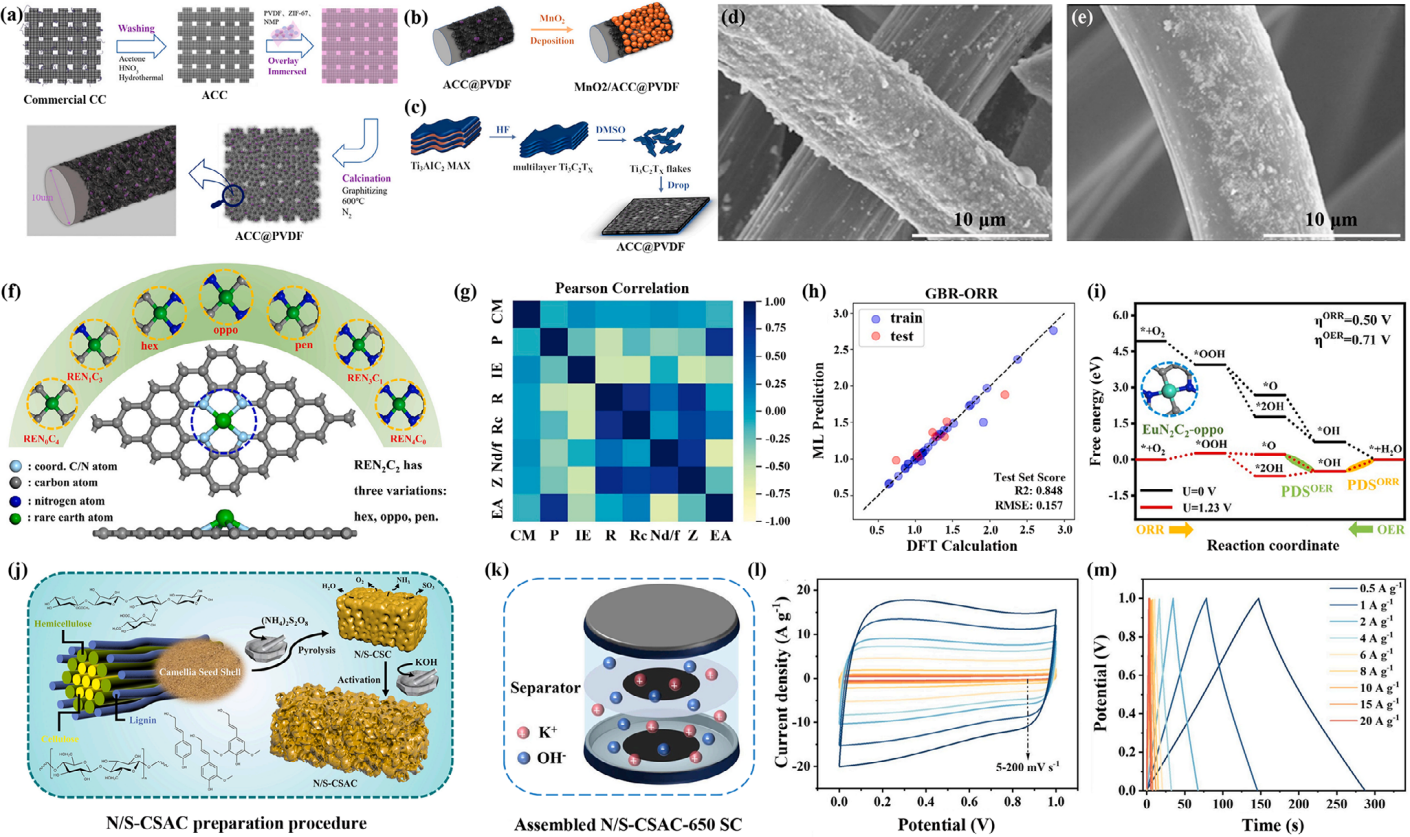
(a) Schematic illustration of the preparation of substrate material ACC@PVDF. (b) Positive electrode MnO_2_/ACC@PVDF. (c) Negative electrode Mxene/ACC@PVDF. (d, e) SEM images of ACC@PVDF0.15 and ACC@PVDF0.05. Reproduced with permission [95]. Copyright 2023, Elsevier. (f) Optimal configurations of RENxC4‐x electrocatalysts. (g) The heat map displaying the Pearson correlation coefficients between the selected features. (h) Comparison between the calculated values of ORR. (i) Free energy reaction profiles for the six catalysts. Reproduced with permission [98]. Copyright 2024, Elsevier. (j) Diagrammatic representation of the N/S‐CSAC preparation procedure. (k) Schematic diagram of the assembled N/S‐CSAC‐650 SC. (l) CV curves of the N/S‐CSAC‐650 SC at 5–200 mV s^−1^. (m) GCD curves tested at 0.5–20 A g^−1^. Reproduced with permission [101]. Copyright 2024, Elsevier.

In a highly valuable study, Liu et al. [95] introduced a flexible self‐supporting nitrogen‐fluorine doped and cobalt‐embedded activated carbon electrode material (ACC@PVDF). First, the impurities on the surface of the carbon cloth were removed by washing and acid treatment, followed by thermal processing to obtain activated carbon cloth (ACC). Then, ACC was mixed with polyvinylidene fluoride (PVDF), and the ACC@PVDF composite material was obtained through a calcination treatment (Figure 6a). The process of depositing manganese dioxide (MnO_2_) on the ACC@PVDF to form the MnO_2_/ACC@PVDF composite material was also demonstrated (Figure 6b). This material could potentially be used to enhance the electrochemical performance of the electrode. Figure 6c shows how the MnO_2_/ACC@PVDF composite material can be applied in flexible electronic devices as electrode material for supercapacitors. Additionally, SEM images display the fibrous surface structure of the ACC@PVDF composite material (Figure 6d), and detail the possible particulate matter on the fiber surface, which may be PVDF or other substances formed during the calcination process (Figure 6e). Overall, this study presents a method for preparing high‐performance carbon‐based electrode materials through modification and composition, and highlights their application potential in flexible electronic devices [96, 97]. Analysis of the SEM images reveals that the modified carbon material has a unique microstructure, which significantly influences the enhancement of its electrochemical properties.

Recently, Fu et al. [98] proposed a quantum chemistry‐based DFT‐ML method that establishes a universal framework for the discovery and design of future materials. The study also elucidates the structure‐property relationships of different rare earth‐modified catalysts in terms of stability, electronic properties, and catalytic activity, providing theoretical guidance for the prediction and synthesis of rare earth‐modified carbon‐based oxygen electrode catalysts. As shown in Figure 6f, different types of carbon nanostructures and their impact on electrode performance are depicted [99]. Moreover, variations in these structures (such as topological structures and porosity) may affect the electrochemical performance of electrode materials. The Pearson correlation coefficient matrix between different variables (Figure 6g) can identify which variables have significant linear relationships, which is very useful for understanding the factors affecting material performance. Furthermore, the comparison between the prediction results based on the Gradient Boosting Regression (GBR‐ORR) model and the density functional theory (DFT) calculation results (Figure 6h) demonstrates the accuracy and fit of the model, and its ability to predict DFT calculation results effectively, verifying the model's validity. Additionally, the free energy diagram describes the changes in free energy for different reaction steps in the oxygen reduction reaction (ORR) process (Figure 6i), and these changes in free energy reveal the reaction pathways and energy barriers [100], which are crucial for understanding the electrocatalytic mechanisms and designing more efficient electrocatalysts.

It is worth noting that Yang et al. [101] designed a new type of flexible self‐supporting N/S co‐doped biomass porous carbon‐based electrode material (N/S‐CSAC‐650) (Figure 6j), through in situ expansion, heteroatom doping, and subsequent KOH activation strategy. This composite electrode exhibits high capacitance, good cycling stability, and excellent mechanical performance, providing a potential solution for the application of future flexible self‐supporting supercapacitors. The assembled N/S‐CSAC‐650 SC supercapacitor includes the layout of electrodes, separator, electrolyte (containing K^+^ and OH^−^ ions), and the outer casing (Figure 6k), a structure that facilitates the transport of ions in the electrolyte [86], thus enabling the charging and discharging cycles of the capacitor. Cyclic voltammetry (CV curves) at different scanning rates is used to evaluate the electrochemical performance of the supercapacitor. By varying the scanning rate from 5 mV/s to 200 mV/s (Figure 6l), it effectively reflects the relevant information about the capacitive characteristics of the capacitor. Additionally, the galvanostatic charge/discharge (GCD) curves at different current densities show the change in potential over time (Figure 6m), which can be used to calculate the specific capacitance and energy density of the capacitor. Overall, the team's research offers a comprehensive perspective on the preparation, assembly, and electrochemical performance testing of a new type of supercapacitor active material [20, 61]. Through these studies, researchers can assess the capacitive performance, rate performance, and working conditions at different current densities of 0.5–20 A g^−1^, which is of great significance for the development of high‐performance electrochemical energy storage devices.

To sum up, in order to more intuitively understand the different spatial and electronic effects caused by various doping atoms (such as N, P, S) and their synergistic effects [91, 92, 93], we have conducted some analysis and summarization on this matter. First, single‐element dopants (N, P, S, B) are treated in sequence. Quantitative results are provided for lattice‐parameter changes, charge‐density differences, and Bader charges [95]. For example, graphitic N lengthens the C─N bond by 0.8% and introduces +0.45 e charge, raising the ORR half‐wave potential by 70 mV (to 0.88 V vs. RHE), and lowering the Tafel slope to 65 mV·dec^−1^. While the P─C_3_ bond expansion of 1.2% creates a electron‐donating/empty‐orbital dual center that reduces the HER over‐potential by 85 mV [96, 101]. Second, co‐doping effects are systematically compared. An N/P donor–acceptor pair decreases the charge‐reorganization energy (*ΔE*) to 0.12 eV and further increases the half‐wave potential by 30 mV, demonstrating synergy, whereas a high P loading (> 5 at %) blocks N active sites and causes a 15% drop in ORR activity [98], revealing antagonism. Finally, three mainstream doping strategies, in situ pyrolysis, post‐treatment with NH_3_/H_2_S, and plasma etching are reviewed, and their differences in doping efficiency, uniformity, and scalability are analyzed.

### 3.2 Surface Functional Modification

Surface functional modification of carbon‐based electrodes is an important means to enhance their electrochemical performance and has attracted widespread attention in recent years. Through surface functional modification, the specific surface area, the number of surface active sites, and the wettability with electrolytes of carbon‐based electrodes can be significantly improved, thereby enhancing their application performance in fields such as supercapacitors and flow batteries [20, 102].

Common methods for surface functional modification include chemical etching, heteroatom doping, and nanomaterial compositing. For example, KOH thermal etching can effectively increase the specific surface area and specific capacitance of CFs, with minimal impact on their mechanical properties [103]. Moreover, by introducing functional groups containing heteroatoms such as nitrogen and oxygen onto the surface of carbon electrodes, their hydrophilicity and electrochemical activity can be enhanced [104]. For instance, CF electrodes modified with aminosilane coupling agents show a significant increase in specific capacity and a substantial improvement in electric field response sensitivity [105].

In the field of flow batteries, surface functional modification also demonstrates great potential. For example, the team led by Song et al. [106] from Xi'an Jiaotong University constructed an oxygen‐rich nanocarbon layer on carbon felt through non‐equilibrium magnetron sputtering combined with heat treatment, which significantly improved the electrochemical performance of vanadium flow batteries [102]. These studies indicate that surface functional modification can not only improve the intrinsic properties of carbon‐based electrodes but also further enhance the overall performance of batteries by optimizing their interactions with electrolytes [107].

In summary, the methods for surface functional modification of carbon‐based electrodes are diverse and effective. Future research directions may include the development of more efficient modification techniques, exploration of novel functional materials, and further optimization of modification processes to meet the needs of different application scenarios [108].

**FIGURE 7:**
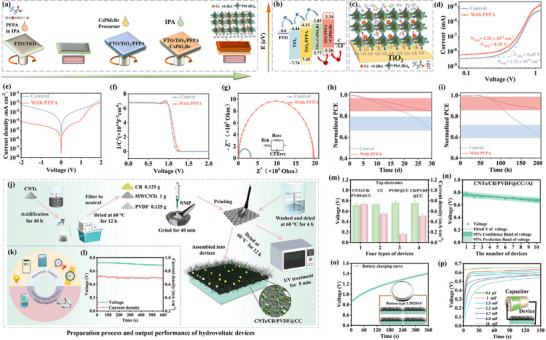
(a) Schematic diagram of TiO_2_ modification and CsPbI_2_Br perovskite films preparation. (b) The schematic energy level arrangement of CsPbI_2_Br perovskite films w/o and with PFPA modification. (c) The mechanism schematic illustration of PFPA modification. (d) SCLC measurement of the control CsPbI_2_Br film and PFPA‐based film with electron‐only device: FTO/TiO_2_/with or without PFPA/CsPbI_2_Br/PCBM/Ag. (e) Dark J–V curves. (f) Mott−Schottky (M−S) plots. (g) Nyquist plots of the control CsPbI_2_Br device and PFPA‐based device. (h) Long‐term storage stability in nitrogen atmosphere. (i) thermal stability of control devices and PFPA‐based devices. Reproduced with permission [109]. Copyright 2024, Elsevier. (j) Preparation process of hydrovoltaic devices. (k) The power generated by the device can either be utilized directly or stored for later use. (l) Output performance of the CNTs/CB/PVDF@CC//Al device. (m) Average performance output of four types of devices. (n) Voltage error bands for 10 devices. (o) V–t curve for charging batteries by hydrovoltaic devices. (p) V–t curve for charging capacitors of different capacities by hydrovoltaic devices. Reproduced with permission [110]. Copyright 2024, Wiley.

It's interesting that Huo et al. [109] developed a “bottom‐up” multi‐interface modification strategy, utilizing perfluoropropionic acid (PFPA) as a surface defect passivator, which simultaneously improved the interface quality between the perovskite and the electron transport layer (ETL) as well as the carbon electrode, significantly enhancing the power conversion efficiency (PCE) and stability of the devices. It illustrates the preparation process of PFPA and the subsequent fabrication of the CsPbI_2_Br perovskite layer on PFPA‐modified FTO via the antisolvent method (Figure 7a). The introduction of PFPA led to a rearrangement of energy levels (Figure 7b), particularly raising the conduction band (CB) position of TiO_2_, reducing the energy level offset with the perovskite, and facilitating electron transfer. The interactions between PFPA molecules and TiO_2_ and perovskite [66, 80], including hydrogen bonding between PFPA and surface oxygen atoms of TiO_2_, as well as coordination between PFPA and lead atoms on the perovskite surface (Figure 7c), are precisely these interactions that help passivate surface defects and improve interface quality. Furthermore, the current density–voltage (J–V) curves of the PFPA‐modified group imply that devices with PFPA modification have higher short‐circuit current density (Jsc), open‐circuit voltage (Voc), and fill factor (FF), thereby achieving a higher power conversion efficiency (PCE) (Figure 7d).

Additionally, the J–V curves imply that the group with PFPA added exhibits higher current density at higher voltages (Figure 7e), indicating superior electrochemical performance. Moreover, the group with PFPA added shows higher capacitance over a wider voltage range (Figure 7f), suggesting better capacitive performance [92]. The EIS demonstrates that the group with PFPA added displays a smaller semicircle, indicating lower charge transfer resistance (R_ct_) and interfacial resistance (R_s_), and thus lower electrochemical impedance (Figure 7g). On the other hand, the group with PFPA added exhibits higher Coulombic efficiency in the short term (Figure 7h), with slower decay, indicating better cycling stability. Concurrently, the group with PFPA added also shows higher Coulombic efficiency in the long term (Figure 7i), with slower decay, further confirming its excellent cycling stability. In summary, batteries with PFPA added have higher current density, greater capacitance, lower electrochemical impedance, and better cycling stability, thereby effectively enhancing the electrochemical performance of the batteries [103, 106].

Liu et al. [110] designed an asymmetric sandwich‐structured hydrovoltaic device, with the top electrode composed of carbon cloth (CC) coated with CNTs [4], the bottom electrode made of an aluminum (Al) plate, and separated by porous filter paper (Figure 7j). The plasma‐treated CNTs/CB/PVDF@CC not only exhibited strong hydrophilicity and enhanced interaction with water but also increased the electrode surface area, improving the device's evaporation rate. Additionally, the device produced a power density of 124.5 µW·cm^−2^ over an area of 1 cm^2^. The J–V curves indicate the output performance of the device under different loads (Figure 7k), with the inset showing a photograph of the device (Figure 7l), illustrating its potential for practical applications. Furthermore, the voltage outputs of four different types of devices were compared, including combinations of top electrodes CC, PVDF@CC, CB/PVDF@CC, and CNTs/CB/PVDF@CC with the bottom Al electrode [38], and the results showed that the CNTs/CB/PVDF@CC//Al combination had the highest voltage output (Figure 7m). A statistical analysis of the voltage output from 10 CNTs/CB/PVDF@CC//Al devices at different numbers of devices, including the mean voltage and 95% confidence interval, demonstrated the consistency and reliability of the voltage output (Figure 7n). The V–t curve for charging commercial button‐type lithium batteries (LIR2016) was analyzed, showing that the device could charge commercial batteries from 0.9 V to 1.4 V (Figure 7o). Additionally, the V–t curves for charging capacitors of different capacities indicated that the device could charge capacitors of 0.1 µF, 1.5 µF, and 4.7 µF (Figure 7p), further proving its versatility and applicability. Overall, the study detailed the preparation process, output performance, and potential for practical application of hydrovoltaic devices, also demonstrating the significant application prospects of such devices in the field of green energy harvesting and conversion.

### 3.3 Plasma‐Based Modifications

Plasma modification of carbon‐based electrodes has graduated from a laboratory second‐scale demo to a production‐line takt‐time process. Its footprints now span sensing, energy and green hydrogen, sketching a technology roadmap that expands point‐wise from the microscale to the tonne scale.

For example, the first breakthrough came with screen‐printed carbon electrodes (SPCE) [111]. A five‐second O_2_‐plasma sweep instantly boosts surface carboxyl density, drops the contact angle and slashes charge‐transfer resistance from 7.1 kΩ to 0.45 kΩ. After antibodies are covalently anchored via EDC/NHS, the IgA immunosensor gains 2.4× sensitivity and batch‐to‐batch RSD shrinks from 9.7% to 0.8%, delivering the final interfacial chemistry needed for million‐piece disposable electrode lots. At 700°C, RF NH_3_ plasma becomes both nitrogen source and etching blade, simultaneously doping and exposing active edges on Co_9_S_8_/graphene composite electrodes [103, 104]. The resulting ORR onset and half‐wave potentials outperform untreated samples, so the same sheet can be die‐cut into PEM fuel‐cell cathodes—one electrode, one reaction, zero extras. Stepping out of the vacuum chamber, roll‐to‐roll atmospheric Ar–H_2_O plasma runs at 120 pieces h^−1^. A 2.1 µm polymer skin on 3D‐printed CB/PLA carbon electrodes is stripped in a flash, pushing the nitroglycerin detection limit from 0.5 µM to 0.1 µM and lifting the signal‐to‐noise ratio 15‐fold. The full print‐treat‐package sequence is now ISO 9001:2015 line‐qualified and rides on a single conveyor.

An even larger scale is handled by low‐temperature NH_3_ plasma: within seconds it treats Ni‐MOF‐derived carbon cloth, embeds nitrogen, and refines nanocrystals into a free‐standing Ni─N─C electrode. Hydrogen evolution overpotential falls below 40 mV at 10 mA·cm^−2^, and 500 kg per batch are already shipped under supply contracts, plasma's first entry into the tonne‐scale catalytic‐electrode market.

From five seconds to sub‐second, from room temperature to 700°C, from single sheets to roll‐to‐roll to tonne‐level catalysis, these cases collectively portray the three‐in‐one allure of plasma on carbon electrodes: doping, cleaning, and roughening in one dry step, while conductivity, active‐site density, and interfacial strength rise in concert. Plasma is no longer the understudy of wet chemistry; it is the metronome for next‐generation electrode manufacturing, high uniformity, low carbon footprint, and high throughput.

### 3.4 Structure Optimization

Generally, the structural optimization of carbon‐based electrodes is key to enhancing their performance in energy storage and conversion devices. By controlling the ratio between types of carbon sources and different components (activators or templates), as well as variable factors such as carbonization temperature/time, it is possible to design and optimize the pore structure and specific surface area of carbon‐based anode materials. This is achieved by utilizing their abundant ion adsorption/desorption channels and active sites to construct high‐performance devices. Surface modification of carbon materials can also be carried out using heteroatoms to regulate the physicochemical properties of the electrode surface, including wettability, conductivity, and electronegativity, thereby improving the electrochemical performance of the device [112]. Additionally, pseudocapacitance reactions with zinc ions can be enhanced through surface functional groups to improve energy storage effects. It is even possible to combine 1D CNTs with 2D graphene nanosheets (GN‐CNTs) to describe a new type of 3D material microbial electrode [113]. This realizes strong bacterial adhesion and proliferation, and the combination of its macroscopic and nanoscopic structures can effectively provide positive charges on the cathode surface, increasing current consumption and the rate of microbial electrochemical synthesis.

**FIGURE 8:**
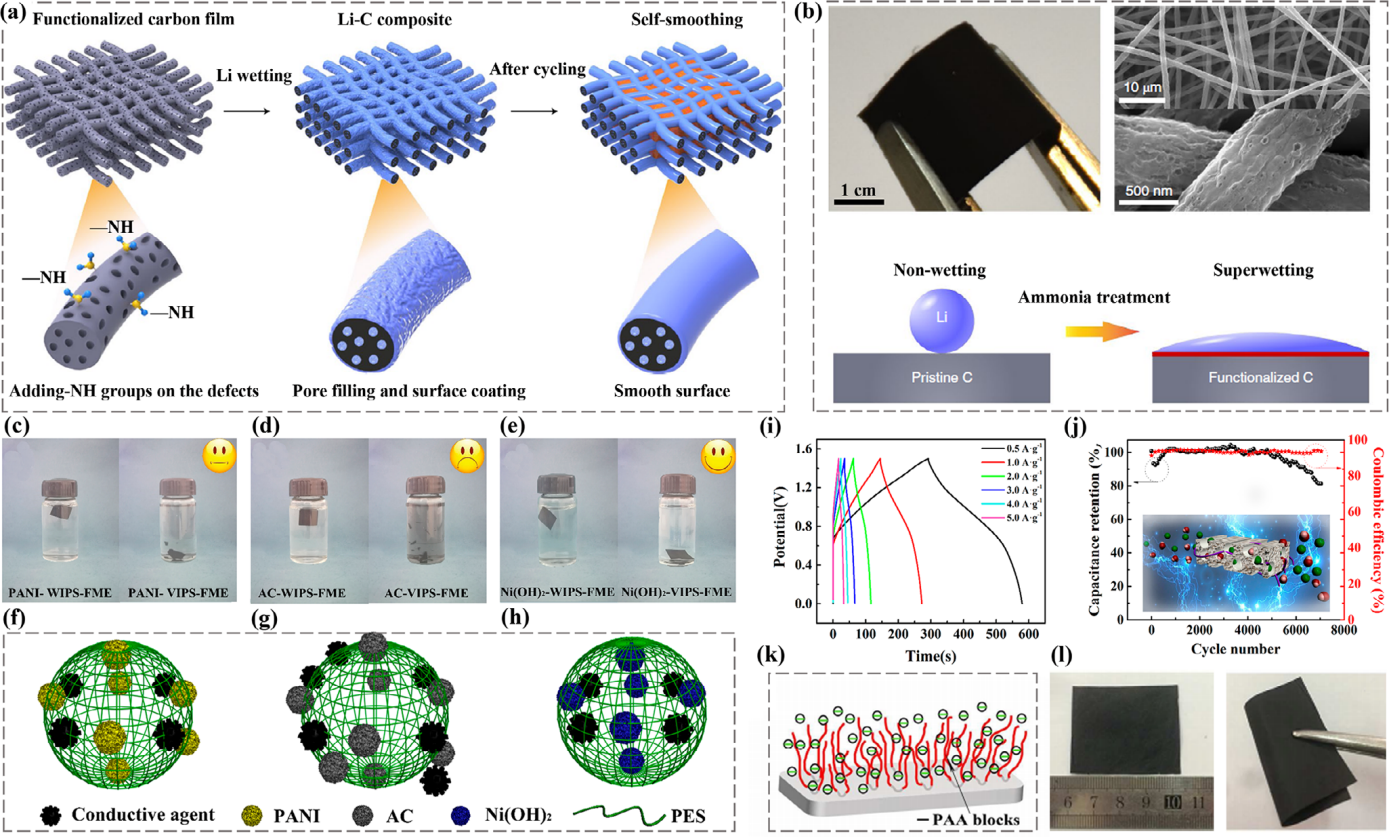
(a) Illustration of self‐smoothing behaviour in the Li─C anode. (b) Characterizations of Li infiltration into carbon film. Reproduced with permission [114]. Copyright 2019, Springer Nature. Physical form of FME composed of different active substances after ultrasonic oscillation of (c) PANI‐WIPS‐FME and PANI‐VIPS‐FME. (d) AC‐WIPS‐FME and AC‐VIPS‐FME. (e) Ni(OH)_2_‐WIPS‐FME and Ni(OH)_2_‐VIPS‐FME. (f−h) Mechanism diagram corresponding to the three membranes. (i) GCD (0.5−5A g^−1^) for VIPS‐FME||AC‐FME ASC device. (j) Cycle life. Reproduced with permission [117]. Copyright 2019, American Chemical Society. (k) Schematic illustration. (l) Photographs of amphiphilic block copolymer modified electrode film. Reproduced with permission [120]. Copyright 2018, The Royal Society of Chemistry.

To illustrate the significance of optimizing the structure of carbon‐based electrodes, Niu et al. [114] developed a self‐smoothing lithium─carbon (Li─C) anode structure based on amine‐functionalized 3D mesoporous CFs for achieving high‐energy‐density lithium metal batteries (LMBs) (Figure 8a). This structure improves the wettability of lithium on the carbon surface [115], promoting uniform lithium deposition and preferred nucleation, thereby achieving reversible and self‐smoothing lithium deposition during cycling. To address the poor wettability of the pristine carbon film, which prevents lithium metal from penetrating the carbon film effectively, ammonia treatment is utilized to functionalize the surface of the carbon film, thereby achieving superwetting, allowing lithium metal to infiltrate and distribute more effectively (Figure 8b). Furthermore, the microstructure of the functionalized carbon film is observed through SEM images, including the diameter of the fibers, and the size of the pores [116], and the analysis of these structural features contributes to the uniform distribution and deposition of lithium. Overall, this study demonstrates that introducing ‐NH groups on the surface of carbon materials can significantly improve the wettability and distribution of lithium metal, thus enabling self‐smoothing lithium deposition, which is of great importance for enhancing the performance and stability of lithium metal batteries.

Additionally, Dong et al. [117] proposed a method for preparing flexible membrane electrodes through a slow phase separation induced by water vapor, with the aim of increasing their energy density by enhancing the specific capacitance of the active material or expanding the potential window [118]. The research found that the VIPS‐FME||AC‐FME hybrid supercapacitor exhibited a maximum energy density of 30.01 Wh·kg^−1^ and a maximum power density of 3750.95 W·kg^−1^. These excellent electrochemical performances were mainly due to the satisfactory symmetrical structure of the membrane electrode. Figures 8c‐e show photographs of different flexible membrane electrodes (FMEs), including PANI, AC, and nickel hydroxide (Ni(OH)_2_)‐based FMEs. These FMEs were prepared by water‐induced phase separation (WIPS) and vapor‐induced phase separation (VIPS) methods, respectively labeled as WIPS‐FME and VIPS‐FME. Moreover, the emoticon symbols in the figures may indicate the difficulty of the preparation process or the level of satisfaction with the results. The schematic diagrams of FME (Figure 8f) include conductive agents, PANI, AC, nickel hydroxide (Ni(OH)_2_), and polyether sulfone (PES). It also shows the porous structure of the electrode material (Figure 8g,h), which aids in the transport of electrolyte ions and improves electrochemical performance [119]. CV curves at different scan rates illustrate the electrochemical behavior and capacitive characteristics of the electrode materials (Figure 8i). The capacitance retention of the electrode after long‐term cycling indicates the cycling stability of the electrode material (Figure 8j).

Another representative study, Wang et al. [120] studied a new type of amphiphilic block copolymer‐modified membrane electrode for supercapacitors. The prepared electrode membrane exhibited high specific capacitance and excellent cycling stability, and the assembled symmetric supercapacitor maintained a capacitance retention rate as high as 99% after 10 000 cycles at a current density of 2 A/g.

In summary, this study provides a simple method for the surface modification of flexible membrane electrodes [121], which contributes to the fabrication of high‐performance flexible supercapacitors. The distribution and ion transport process of polyacrylic acid (PAA) blocks within the electrode material (Figure 8k) are illustrated, showing the migration and distribution of ions in the electrode material over time, which may be related to the electrochemical performance of the electrode. However, this ion transport is crucial for the performance of energy storage devices such as supercapacitors [20, 101], as it affects the efficiency of charge storage and release. Additionally, the practical application of the flexible membrane electrode is demonstrated, where the flexible membrane electrode does not get damaged when folded or bent, indicating its good flexibility and durability (Figure 8l).

### 3.5 Carbon‐Based Composite Design

Carbon‐based composites are designed using carbon materials (graphite, graphene, CNTs, hard carbon, porous carbon, CFs, etc.) as the continuous phase [122−125]. Through multiscale structural manipulation and coupling with functional components, they create electrode systems that combine high conductivity, high stability, and highly active interfaces. The core strategy can be summarized as a 3D hierarchical porous framework, defect/heteroatom active sites, and enhanced interface coupling [126]. First, a hierarchical interconnected macropore‐mesopore‐micropore structure is constructed using templating, activation, electrospinning, or 3D printing to shorten ion migration paths and mitigate volume expansion. Subsequently, heteroatom doping with nitrogen, sulfur, phosphorus, and boron, or the introduction of topological defects, modulates the electronic structure of the carbon layer, generating pseudocapacitance or catalytically active centers. Furthermore, metal oxides, sulfides, single‐atom catalysts, or conductive polymer nanodomains are uniformly anchored on the carbon framework surface and within its pores, forming strong interfacial bonds that inhibit particle aggregation and promote charge transfer. Simultaneously, the integrated design of the binder and current collector is optimized to achieve a seamless connection across the entire electrode conductive network. Advanced in situ characterization and high‐throughput computing assisted by machine learning are accelerating the decoding of microstructure‐performance relationships, enabling carbon‐based composites to achieve specific capacitance exceeding 1000 F/g, 10 A/g rate capability [127], and 10 000 cycle life in scenarios such as supercapacitors, lithium/sodium/potassium ion batteries, lithium‐sulfur batteries, and zinc‐air batteries.

Carbon‐based composite battery design toward 2025 demonstrates parallel advances across multiple electrochemical systems [128]. In lithium‐ion batteries, breakthroughs include a 3D nitrogen‐doped graphene/sulfur cathode with 68 Wh/kg energy density and 98% retention over 5000 cycles [129], carbon‐coated LiFePO_4_ nanoparticles delivering near‐theoretical capacity of 167 mAh/g at 0.1 C [130], and a porous C@MoS_2_─Sn anode achieving 841 mAh/g—more than twice that of graphite [131]. Redox flow batteries benefit from carbon fiber–expanded graphite–vinyl ester bipolar plates with ultra‐low resistance of 6 mΩ·cm^2^ and high flexural strength of >50 MPa, enabling 30% lighter kilowatt‐scale stacks for cost‐effective long‐duration storage. Metal–air batteries gain durability from Ru‐decorated, TiC‐coated CNT frameworks, extending Li─O_2_ cycle life fourfold, while microbial fuel cells achieve 55% higher power density and shortened startup times using ammonia–acid treated CNT/CC anodes.

To reconcile performance with stability, AI‐driven inverse design has yielded a carbon–aluminum gradient anode capable of tolerating 300% volume expansion while sustaining >1000 mAh/g with <0.05% capacity fading per cycle [132]. Meanwhile, sustainable processing strategies, such as one‐step carbonization of bio‐based precursors coupled with microwave expansion, reduce electrode fabrication energy consumption by 40% [133], offering a scalable and environmentally responsible route to next‐generation energy storage.

In summary, carbon‐based composite design has achieved significant breakthroughs in strength, functionality, and green manufacturing. For example, aviation‐grade T1100G/M40X CF paired with 3960 low‐temperature epoxy achieves a 29% increase in strength and an elongation of >1.5% through nanographite orientation manipulation and covalent‐noncovalent synergistic interface modification [134]. Rapid curing in 5–10 min using RTM has been used in drones. In the field of electromagnetic wave absorption, core‐shell structures such as CNT@MnO_2_ and CS@MoS_2_ achieve reflection losses ←50 dB in the X‐Ku band and bandwidths >5 GHz through multipolarization and gradient impedance [135]. The carbon shell protects the magnetic core and provides resistance to marine corrosion. Paper‐based flexible composites have an areal density of 30 g/m^2^ and a conductivity >1000 S/cm. Thermoplastic TPU‐CNT films, which can be heated from a low voltage of 5 V to 100°C in 20 seconds, have been used in wearable heating applications [136]. CFRP rail transit bodies have been reduced in weight by 30% and have passed 10^7^ cycles of fatigue testing. The thermal conductivity of carbon/carbon composites for spacecraft applications has exceeded 800 W/(m·K), and the ablation rate of ZrC‐SiC coatings at 2000°C in an oxygen environment is less than 0.3 mg/(cm^2^·s) [137]. AI potential functions and high‐throughput computing have halved R&D cycles. Bio‐based resins and solvent‐free RTM have reduced lifecycle carbon emissions by 40%, moving toward next‐generation green, low‐carbon materials that integrate structure, function, and intelligence.

### 3.6 Theoretical‐Calculation Assisted Optimization of Electrode Performance

It is noteworthy that the core of the multifunctional integration strategy lies in the active modulation of the intrinsic electronic properties of carbon‐based materials through doping, defect, and interface engineering. At this level, DFT calculations play an indispensable role as an atomic‐scale probe [138]. Specifically, for heteroatom doping (e.g., N/S co‐doping), calculations of the density of states and Bader charge clearly reveal how doping disrupts the charge neutrality of the carbon framework, introduces asymmetric charge distribution, and modulates the Fermi level position. This charge redistribution directly creates active sites with optimal adsorption strength for reaction intermediates (such as O_2_ in ORR, and Li_2_S in Li‐S batteries) [26, 27], fundamentally explaining the synchronous enhancement of conductivity and catalytic activity. For defect engineering, analysis of the local density of states visualizes the localized electronic states at defects (e.g., vacancies, edges). These highly active electronic states are key to accelerating surface reaction kinetics. Simultaneously, calculating the migration energy barrier of lithium ions near defects using the climbing image nudged elastic band method provides direct quantitative predictions for rate performance.

In summary, deeply integrating theoretical tools such as DFT into carbon‐based electrode research marks a transition in our work from phenomenon observation and performance listing to mechanism‐driven and rational design [98, 99]. It transforms our proposed conductivity‐stability‐activity ternary coupling matrix from an abstract concept into a physical model supported by concrete electronic structure insights and quantitative descriptors. Looking ahead, this mechanistic understanding and integrated design dual‐drive research framework can be combined with machine learning potentials and high‐throughput calculations to achieve efficient screening of vast candidate materials and configurations. Ultimately, this will enable a leap from single‐point optimization and synergistic design to systematic creation, laying a solid theoretical foundation for developing next‐generation high‐performance energy storage materials.

Finally, to evaluate the relative advantages and limitations of various carbon‐based electrode modification strategies, including elemental doping, surface functionalization, plasma treatment, structural optimization, carbon‐based composite design, and theory‐guided design, in terms of performance metrics and mechanisms, we have systematically compiled a comparative table outlining the key strengths and weaknesses of major modification approaches (Table 1) [95, 109, 111, 114, 129, 138]. This table aims to provide researchers with a clear decision‐making framework and a valuable reference for academic writing.

**TABLE 1:** Comparison and analysis of carbon‐based electrode modification strategies.

Modification strategy	Core mechanism	Key advantages	Main limitations	Representative performance metrics examples
Elemental doping (e.g., N, B, S, P) [95]	Introduces heteroatoms to break charge neutrality; Modulate charge distribution and band structure; Create active sites	Effectively enhances conductivity; Creates catalytic active sites; Improves ion adsorption; Relatively mature methodology	Difficulty in precise control of doping concentration and configuration; may introduce unwanted defects; Single doping effect often has bottlenecks	Conductivity increased by 3–10 times; ORR half‐wave potential positively shifted by 20–70 mV; ion adsorption energy optimized by 0.1–0.5 eV.
Surface functionalization (–COOH, –OH, –C=O, etc.) [109]	Introduces O/N–containing functional groups to alter surface chemistry, wettability, and reactivity	Significantly improves hydrophilicity/wettability; Provides pseudocapacitance;Serves as anchor points for further modification; Simple process	Excessive functionalization severely degrades conductivity; Functional groups may be unstable during cycling; May trigger side reactions	Contact angle reduced by >30°; contributes 50–200 F/g of pseudocapacitance in aqueous electrolytes; but initial Coulombic efficiency may drop by 5–15%.
Plasma‐based (Plasma etching/nitridation, etc.) [111]	Uses energetic particle bombardment to simultaneously introduce defects, doping, and functional groups on the surface	Efficient, clean, chemical‐free; Can treat complex structures deeply and uniformly; strong synergistic effects.	Expensive equipment, high processing cost; Process parameters are highly sensitive; Compromising mechanical strength.	Specific surface area can increase by 20%–100%; surface N/O atomic concentration increases by 5–15 at.%; rate performance significantly improves.
Structural optimization (Hierarchical pores, 3D network, oriented growth) [114]	Designs material morphology and pore structure at micro/nano scales to optimize mass transport paths and stress distribution.	Shortens ion/electron transport paths; Provides ample reaction interface and active site exposure; Inherently high specific surface area.	Poor controllability in synthesizing complex structures; Excessively high specific surface area may lead to low tap density and more side reactions	Areal capacity increased by 2–5 times; capacity retention >80% at 10C rate; capacity fade <0.01% per cycle over 1000 cycles.
Carbon‐based composite structure design (Composite with metal oxides, sulfides, polymers, etc.) [129]	Constructs heterogeneous interfaces to leverage component advantages, generating synergy	Maximizes component strengths; Carbon framework effectively confines active material	Interface contact quality is critical; Composite process is complex; Trade‐off often needed between active material content and electrode performance	Overall gravimetric capacity increased by 50%–300% after compositing; effectively suppresses polysulfide shuttling (Coulombic efficiency >99%).
Theoretical assisted design (DFT, Machine Learning, etc.) [138]	Uses simulations to reveal structure‐property relationships at atomic/electronic scales, predict performance	Provides fundamental mechanistic explanations; Enables high‐throughput virtual screening; Accurately predicts key descriptors	Computational models are simplifications of reality; Difficult to perfectly simulate real complex environments (e.g., electrolyte, SEI film).	Can predict formation energy of doping/defect sites (error <0.5 eV); predicted ion diffusion barriers deviate from experimental values by <0.1 eV

## 4 Applications of Carbon‐Based Electrodes

### 4.1 Energy Storage Technologies and Devices

Notably, carbon‐based electrodes have become the core materials for energy‐storage devices such as supercapacitors and ion batteries owing to their high electrical conductivity, excellent chemical stability, and highly tunable structures [7, 8, 9, 10, 20]. Recent advances have achieved breakthroughs by simultaneously addressing structure, interface [77], and mechanism.

Structurally, nanoconfinement carbonization has produced high‐density porous carbons that deliver record gravimetric of 453 F/g and volumetric of 353 F/cm^3^ capacitances for Zn‐ion hybrid supercapacitor cathodes. Meanwhile, a 3D honeycomb‐like graphene/carbon nanosheet network simultaneously creates fast ion‐transport pathways and mechanical flexibility, enabling flexible micro‐supercapacitors that retain almost 100% of their initial capacitance after 10 000 bending cycles. At the interface, a covalent bonding electrode/electrolyte strategy has been proposed to overcome poor solid–solid contact in solid‐state batteries [139]. Introducing a covalently bonded interlayer between the carbon cathode and the solid electrolyte reduces interfacial resistance by one order of magnitude and triples cycle life. Mechanistically, in situ Raman and in situ quartz crystal microbalance techniques have, for the first time, revealed a cooperative coadsorption–reversible chemisorption mechanism for Zn^2+^ storage in carbon pores [140], clarifying that pore sizes between 1.2 and 5.5 nm are critical for high‐capacity zinc storage. Furthermore, coal‐ and biomass‐derived precursors, processed by hierarchical porosity, heteroatom doping (N, S, P) [86, 91], and defect engineering, have enabled scalable, low‐cost production of high‐performance carbon electrodes, laying a solid material foundation for next‐generation energy‐storage devices with high energy, high power, and long life.

#### 4.1.1 Supercapacitor

**FIGURE 9:**
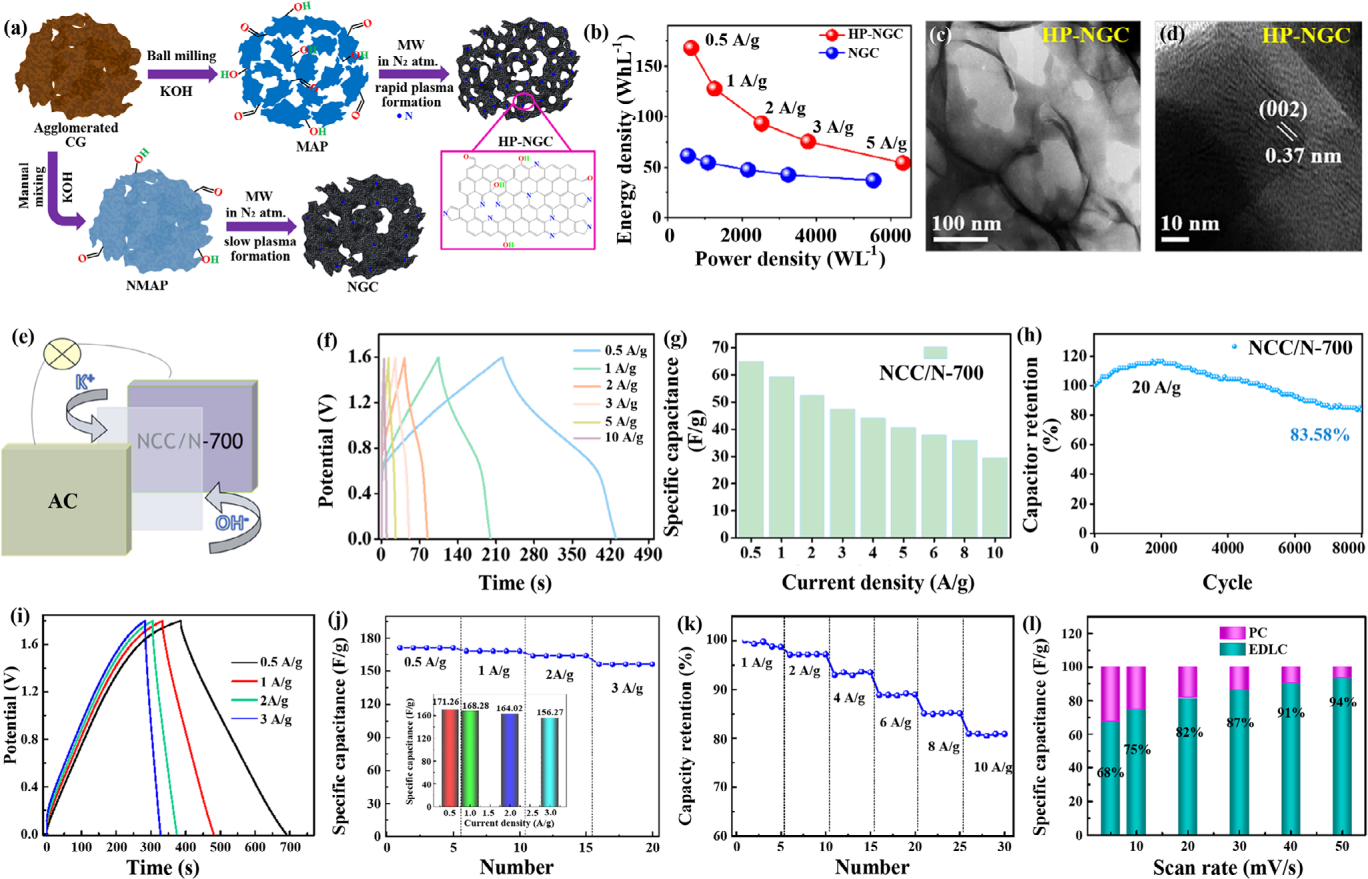
(a) Schematic illustration of the synthesis of HP‐NGC and NGC. (b) Ragone plots of HP‐NGC and NGC electrodes. (c, d) TEM and HR‐TEM images of HP‐NGC. Reproduced with permission [141]. Copyright 2024, American Chemical Society. (e) Schematic diagram of the dual electrodes. (f) GCD curve. (g) Capacitance retention at different current densities for ASC. (h) Cycling performance at 20 A/g of the NCC/N‐700 electrode. Reproduced with permission [142]. Copyright 2025, Elsevier. (i) GCD curves at working potential windows from 0 to 1.8 V. (j) The specific capacitance at various current densities. (k) Rate performance. (l) Capacitance contributions at different scan rates. Reproduced with permission [143]. Copyright 2024, Elsevier.

Choudhury et al. [141] proposed a rapid synthesis strategy termed “mechanochemical‐assisted microwave‐induced nitrogen plasma” to prepare nitrogen‐doped hierarchical porous graphitic carbon (HP‐NGC) using waste coffee grounds as the precursor (Figure 9a), which was subsequently applied as electrodes for aqueous symmetric supercapacitors. The study revealed that ball milling effectively reduced precursor agglomeration and increased oxygen‐containing polar groups, thereby facilitating instantaneous plasma formation and yielding a more uniform porous structure. The resulting HP‐NGC delivered a volumetric capacitance of 630 F/cm^3^ (300 F/g), an energy density of 32 Wh/L, and a power density of 640 W/L (Figure 9b). Moreover, it exhibited outstanding rate performance and retained 79% of its capacity after 10 000 cycles. TEM and HRTEM images in Figure 9c, d confirm the porous morphology of HP‐NGC and the presence of graphitic (002) lattice fringes with an interlayer spacing of 0.37 nm. Overall, this method is environmentally friendly, time‐efficient, and easily scalable, and the obtained HP‐NGC outperforms most reported biomass‐derived carbons in terms of volumetric energy density and cycling stability, offering a new pathway for the development of low‐cost, high‐performance aqueous supercapacitors.

As shown in Figure 9e, the configuration of an asymmetric supercapacitor (ASC) assembled with NCC/N‐700 as the positive electrode and AC as the negative electrode [142]. The galvanostatic charge/discharge (GCD) profiles in Figure 9f, which displays nearly triangular shapes at different current densities, indicating ideal capacitive behavior. Corresponding specific capacitance values as a function of current density are shown in Figure 9g, confirming favorable rate capability. Long‐term cycling stability is demonstrated in Figure 9h, where NCC/N‐700 retains 83.58% of its capacitance after 10 000 cycles at 20 A/g.

In another representative study, Chu et al. [143] employed natural asphalt as the precursor and synthesized hard carbon porous carbon (HCPC‐2) via a one‐step KOH activation–carbonization process. The material exhibited a high specific surface area of 2334 m^2^/g, an interlayer spacing of 0.391 nm, and an oxygen content of 18%. In a three‐electrode system, it delivered a specific capacitance of 326 F/g at 0.5 A/g. When assembled into a PVA–KOH solid‐state symmetric supercapacitor, it achieved a stable operating voltage of 1.4 V, an energy density of 18.9 Wh/ kg, and a power density of 3500 W/kg, with an equivalent series resistance of only 0.82 Ω. The device retained more than 98% of its capacity after 2500 cycles and maintained 71% of its voltage after 72 h of self‐discharge.

The CV curves in Figure 9i maintain quasi‐rectangular shapes across various scan rates, further validating capacitive characteristics. The specific capacitance retention over multiple cycles is summarized in Figure 9j, while extended cycling at different current densities is illustrated in Figure 9k, both highlighting excellent electrochemical durability. Finally, it presents the capacitance contribution analysis at different scan rates (Figure 9l), revealing a hybrid storage mechanism where electric double‐layer capacitance (EDLC) coexists with pseudocapacitance (PC), with the latter contribution increasing at higher scan rates. In a word, this simple and low‐cost process provides a new carbon electrode strategy for high‐performance solid‐state supercapacitors.

#### 4.1.2 Lithium‐Ion Battery

Carbon‐based electrodes have consistently served as the foundation and core of lithium‐ion battery (LIB) technology evolution [22, 31]. In the past three years, research efforts addressing the capacity, cycling stability, rate capability trade‐off have advanced from relying on single graphite to exploring multi‐component synergies among graphite, silicon, and hard carbon [30, 31, 32, 33].

First, the theoretical capacity of conventional graphite anodes has nearly reached its ceiling at 372 mAh/g. To surpass this limit, researchers have expanded the interlayer spacing from 0.335 to 0.37–0.40 nm through oxidation, fluorination, or phosphorus doping, while introducing defects and functional groups [144, 145]. These modifications not only enhance Li^+^ diffusion coefficients but also effectively suppress lithium plating. In 2024, phosphorus–fluorine co‐doped graphite was reported to maintain 350 mAh/g at a 5 C rate, with 92% capacity retention after 500 cycles and an improved initial coulombic efficiency (ICE) of 94% [146], highlighting the promise of deep modification strategies.

Second, silicon–carbon composite anodes have become the focal point for achieving high capacities. Silicon offers a theoretical capacity of up to 4200 mAh/g, but suffers from ∼300% volume expansion that leads to pulverization and rapid failure [147]. The prevailing strategy involves embedding micro/nano‐silicon within carbon frameworks. For instance, a hierarchical porous Si@CNT@C trilayer architecture has achieved 3500 mAh/g at 0.2 A/g with 90% retention after 100 cycles, while watermelon‐like Si/C microspheres retained 80% of capacity at 5 C and reached an ICE of 89% [148]. Here, the carbon matrix not only buffers the mechanical stress of silicon but also establishes a 3D conductive network, acting as a dual‐function buffer–conductor medium for the commercial viability of silicon.

Regarding the aspect of hard carbon anodes, owing to their disordered turbostratic structure and enlarged interlayer spacing of 0.37–0.40 nm, they demonstrate remarkable advantages under fast‐charging and low‐temperature conditions. In 2024, hard carbon derived from natural asphalt via one‐step KOH activation achieved a high surface area of 2334 m^2^/g with hierarchical porosity [143, 149], delivering a reversible capacity of 326 mAh/g at 0.5 A/g. When applied in full cells, it provided an energy density of 18.9 Wh/kg with 98% retention after 2500 cycles and an ICE exceeding 90%. Its low cost and high stability suggest that hard carbon could complement graphite to form a low‐/high‐temperature synergy, broadening LIB application scenarios.

Solid‐state interfacial engineering has further addressed challenges such as low ICE and parasitic interfacial reactions [150]. A pre‐lithiation, interface coating, binder integrated strategy has been proposed: pre‐lithiation with lithium foil increased ICE from 75% to 93%, a 2 nm Al_2_O_3_ layer deposited by atomic layer deposition (ALD) suppressed electrolyte decomposition, and the use of PVDF‐HFP binders with matched elastic modulus limited capacity fading to less than 5% after 500 cycles.

From a process and sustainability perspective, electrospun 1D carbon nanofiber networks have shown excellent mechanical–electrochemical synergy in flexible batteries [96, 151], maintaining 95% capacity after 1000 bending cycles. Moreover, low‐cost precursors such as waste Al─Si alloys and natural asphalt have been employed to prepare carbon–silicon composites [152], balancing resource recycling with cost reduction, and offering practical pathways toward next‐generation high‐energy‐density, low‐cost LIBs.

In summary, carbon‐based electrodes are achieving a new balance among capacity, cycling stability, rate capability, and cost through the coordinated design of microstructure, interface, and processing. This progress is propelling LIBs toward higher energy density, faster charging, and longer cycle life.

#### 4.1.3 Al‐Ion Battery

Al‐ion battery (AIB) uses metallic aluminum as the anode [153, 154], where Al^3+^ ions undergo reversible intercalation/deintercalation between the electrodes to achieve energy storage. Their notable advantages include the high abundance and low cost of aluminum, along with a theoretical volumetric capacity of ∼8 Ah·cm^−3^, which is four times that of lithium. Moreover, aluminum anodes are resistant to dendrite formation, enabling ultrafast charge/discharge (<5 min) and long cycle life exceeding 7000 cycles [155]. The electrolytes are typically ionic liquids, which are non‐flammable, non‐explosive, and operable across a wide temperature range (−40°C to 120°C), ensuring high safety. However, the technology is still in the transition from laboratory research to pilot‐scale application [156]. Its primary drawbacks include a low working voltage of only 1.2–2.0 V, resulting in lower energy density compared to lithium‐ion batteries. In addition, the high charge density of Al^3+^ leads to poor reversibility of cathode intercalation and rapid capacity fading. Ionic liquids also present challenges due to their high cost and strong corrosivity, requiring compatibility with low‐cost separators and packaging. Future research directions focus on the development of high‐voltage cathodes, low‐viscosity electrolytes, and aluminum deposition regulation strategies to achieve low‐cost, long‐life energy storage applications.

**FIGURE 10:**
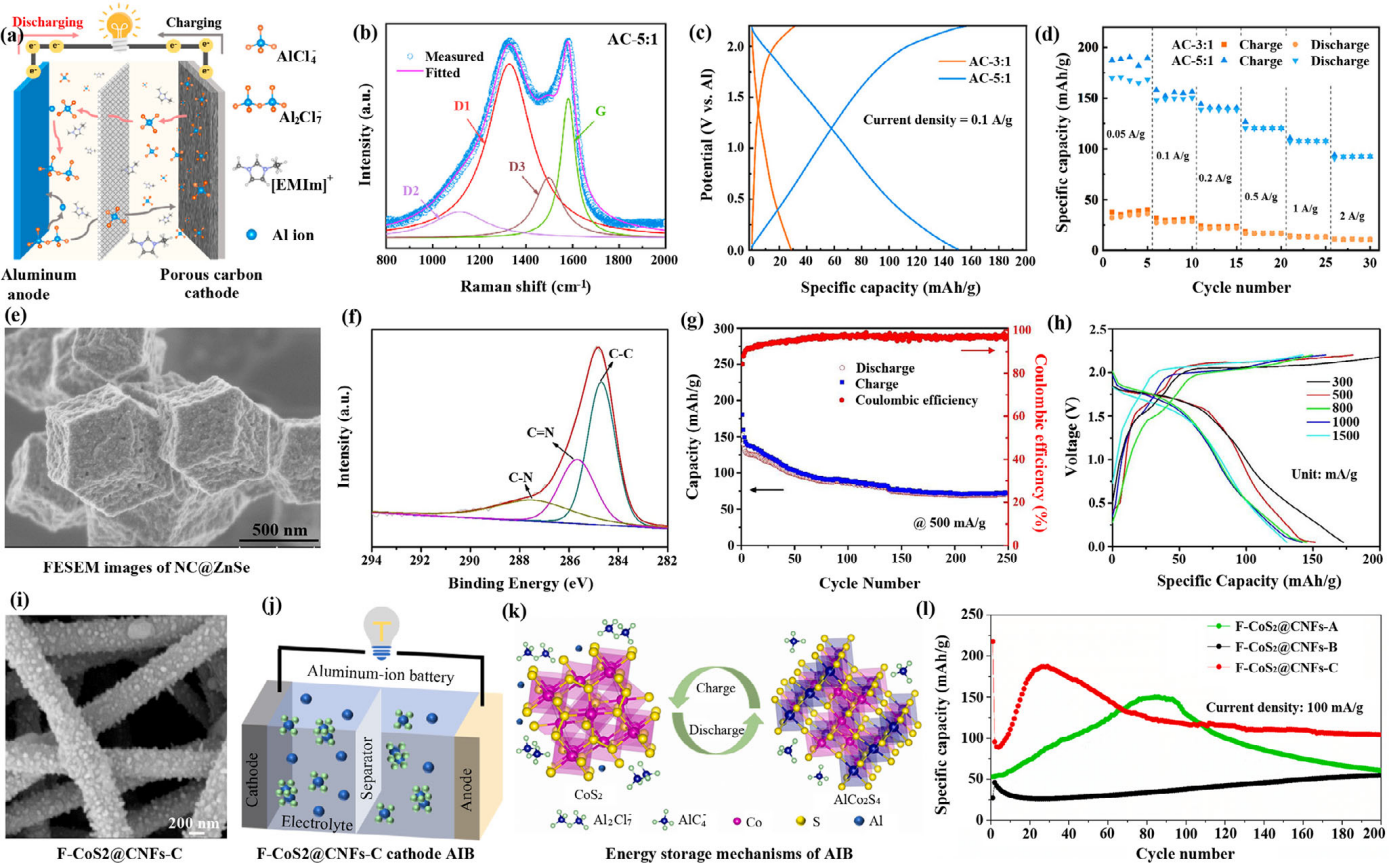
(a) Schematic illustration of AlCl_4_^−^ ion storage by activated carbon cathodes in a non‐aqueous AIB. (b) Deconvolution of D and G bands. (c) Galvanostatic charge/discharge (GCD) curves. (d) Rate capability. Reproduced with permission [157]. Copyright 2022, Elsevier. (e) FESEM images of NC@ZnSe. (f) XPS high resolution spectra of C 1s. (g) Cycling performance and the Coulombic efficiency at 500 mA·g^−1^. (h) Corresponding charge/discharge curves at different current densities. Reproduced with permission [158]. Copyright 2022, Elsevier. (i) SEM images of F‐CoS_2_@CNFs‐C. (j) F‐CoS_2_@CNFs‐C cathode AIB. (k) Its energy storage mechanisms. (l) Cycling performance of AIBs with different binder‐free cathodes. Reproduced with permission [159]. Copyright 2021, Elsevier.

As shown in Figure 10a, it presents the schematic configuration of an AIB with an Al metal anode, a porous carbon‐based cathode [157], and an AlCl_3_/ [EMIm]Cl ionic liquid electrolyte, highlighting the charge/discharge process. The Raman spectrum of AC‐5:1 (Figure 10b), which reveals distinct D and G bands, is indicative of defect density and graphitization degree, while the charge–discharge voltage profiles at 0.1 A/g confirm the reversible intercalation/deintercalation of Al species (Figure 10c). As shown in Figure 10d, AC‐5:1 delivers higher capacity and improved cycling stability compared to AC‐3:1. Additionally, the FESEM image demonstrates the layered porous morphology of NC@ZrSe_2_/C (Figure 10e), which facilitates ion diffusion, and the high‐resolution C 1s XPS spectrum in Figure 10f, it identifies C─C, C─N, and C─O bonds, confirming the incorporation of heteroatom functional groups [158]. And it highlights the long‐term cycling stability at 500 mA/g with stable discharge/charge capacities and nearly 100% coulombic efficiency (Figure 10g), while the rate performance in Figure 10h shows good capacity retention at increasing current densities. The SEM image displays the interconnected nanofiber network of F‐CoS_2_@CNFs‐C (Figure 10i), providing high surface area and conductive pathways, and it illustrates the assembly of an AIB using this composite as the cathode (Figure 10j). The proposed energy storage mechanism involves a combination of AlCl_4_^−^ intercalation/deintercalation and pseudocapacitive surface reactions (Figure 10k). Finally, it compares the cycling stability of different electrodes at 120 mA/g (Figure 10l), demonstrating that F‐CoS_2_@CNFs‐C delivers superior capacity retention and durability [159].

#### 4.1.4 Sodium‐Ion Battery

Sodium‐ion batteries (SIBs) are regarded as a promising alternative to lithium‐ion batteries owing to the abundance and low cost of sodium (Na) resources [160], as well as their compatibility with low‐cost, cobalt‐ and nickel‐free cathode materials such as layered oxides, polyanionic compounds, and Prussian blue analogues. They also employ NaPF_6_–ester electrolytes that can be readily adapted to existing Li‐ion production lines, maintain operation at −20°C [161], and exhibit high intrinsic safety without dendritic sodium deposition. Nevertheless, SIBs still suffer from challenges including significant cathode volume expansion due to the large Na^+^ radius, limited cycle life (2000–5000 cycles), the need for hard carbon anodes with relatively low initial coulombic efficiency of 70%–85%, modest operating voltages of 2.8–3.2 V, and relatively low energy density of 120–160 Wh/kg, only 60–80% of lithium iron phosphate [162]. Furthermore, their rate capability under low‐temperature conditions remains unsatisfactory. Future progress will rely on the development of high‐voltage cathodes, modification of hard carbon anodes, tailored electrolyte additives, and solid‐state designs to close the performance gap with lithium‐ion batteries.

**FIGURE 11:**
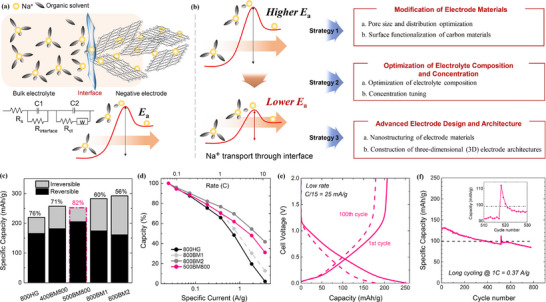
(a) Schematic illustration of the Na ion transfer at the negative electrode/electrolyte interfaces. (b) Schematic illustration of strategies to improve the kinetics of interfacial Na ion transfer. Reproduced with permission [163]. Copyright 2024, Wiley. (c) Comparison of the (ir) reversible specific capacities of the samples, mechanically treated at various stages in sodium half‐cells with 1 M NaClO_4_ in EC:PC electrolyte. (d) Rate capability comparison of the various PVC‐derived soft carbons obtained via mechanical treatment (manual ground, HG or ball‐milled, BM) of the final/intermediate residue against sodium counter electrode and in 1 M NaClO_4_ in EC:PC electrolyte. (e) Cycling performance of the optimized sample 500BM800 vs. Na at a rate of C/15. (f) Capacity retention of 500BM800 at 1C over long cycling‐the inset shows a magnified profile where the Na counter electrode and the electrolyte were replaced with fresh ones at the 518th cycle. Reproduced with permission [166]. Copyright 2022, Elsevier.

Some typical studies in this area, Tsujimoto et al. [163] conducted a systematic review of Na^+^ transport kinetics at the interfaces between carbon anodes, including hard carbon (HC), carbon nanospheres (CNS), and graphite‐like graphene (GLG), and the various organic or polymer electrolytes (Figure 11a). By integrating experimental and theoretical insights into activation energy (E_a_), charge transfer resistance (R_ct_), SEI composition, and pore structure, they demonstrated that the interfacial bottleneck for Na^+^ transport is not solely determined by desolvation, but is also strongly influenced by both the SEI (particularly NaF) and the intrinsic carbon microstructure such as interlayer spacing, closed versus open pores [164, 165]. To address these limitations, they proposed a pore structure–SEI–electric field synergistic regulation strategy, which effectively reduces E_a_ (from ∼70 to ∼50 kJ/mol) and thereby improves both rate capability and long‐term cycling stability (Figure 11b).

In another representative study, to tackle the fundamental trade‐off in soft carbon anodes between achieving high rate capability and maintaining a low initial coulombic efficiency (ICE), Pendashteh et al. [166] developed a distinctive “critical‐point ball milling, re‐passivation” strategy (Figure 11c). In this approach, inexpensive PVC was employed as the carbon precursor, and the carbonization process was intentionally interrupted at 500°C, corresponding to the transition from the molten phase to the onset of solid tar formation. At this critical stage, the intermediate residue was subjected to ball milling to refine particle size [167], after which heating was resumed to 800°C to complete pyrolysis (Figure 11d). This two‐step design leverages the subsequent high‐temperature treatment to promote re‐graphitization and passivation of the freshly exposed surfaces. As a result, the final carbon particles simultaneously achieve a fine size on the order of ∼5 µm and a low specific surface area of 5.8 m^2^/g (Figure 11e, f), effectively mitigating excessive SEI formation and thereby enhancing electrochemical performance.

### 4.2 Electrocatalysis

#### 4.2.1 Fuel Cell

Fuel cells directly convert the chemical energy of hydrogen or hydrogen‐rich fuels into electricity, achieving an efficiency of 50%–60%, which can exceed 80% when combined with waste heat utilization [168]. Their only byproduct is water, making them nearly carbon‐free and noise‐free. It also offers high power density, long driving range, and rapid refueling within just a few minutes, making them particularly suitable for long‐distance applications such as heavy‐duty trucks and ships [169]. However, the current infrastructure for hydrogen production, storage, and transportation remains underdeveloped, while the cost of green hydrogen is still relatively high. In addition, the use of expensive platinum catalysts and proton exchange membranes increases system cost, and durability requires further improvement. Fuel cells are also highly sensitive to hydrogen purity as well as temperature and humidity conditions, posing challenges in cold‐start capability, long‐term stability, and safety management [170]. Moreover, the generation of water can lead to freezing at low temperatures and corrosion under high humidity. In summary, fuel cells hold broad prospects for zero‐emission transportation and distributed power generation, but cost reduction, lifetime extension, and the establishment of a complete hydrogen supply chain are essential for their large‐scale commercialization.

**FIGURE 12:**
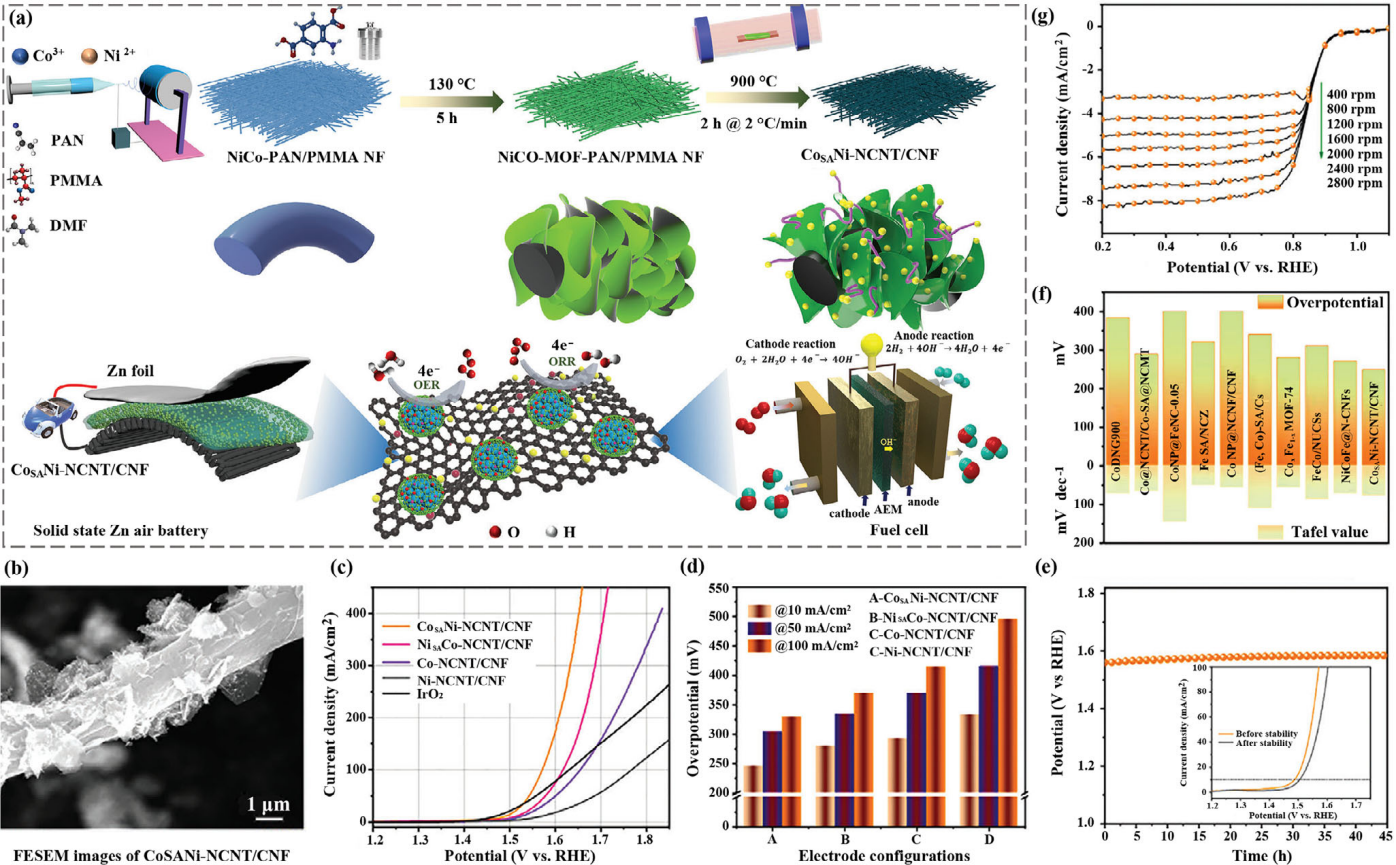
(a) Synthetic scheme for the preparation of Co_SA_Ni‐NCNT/CNF and its application for solid state zinc air battery and fuel cell. (b) FESEM images of Co_SA_Ni‐NCNT/CNF. (c) LSV polarization curves of Ni‐NCNT/CNF, Co‐NCNT/CNF, Ni_SA_Co‐NCNT/CNF, and Co_SA_Ni‐NCNT/CNF. (d) Overpotential at different current densities. (e) Chronopotentiometric profiles of Co_SA_Ni‐NCNT/CNF for 45 h at 10 mA·cm^−2^. (f) Comparison of overpotential and tafel value among series of Co and Ni‐based catalysts in recent literature, ORR performance. (g) LSV curves of Co_SA_Ni‐NCNT/CNF at several rotating speed. Reproduced with permission [171]. Copyright 2024, Wiley.

Poudel et al. [171] employed atomic‐scale engineering to construct a novel 3D self‐supporting bifunctional oxygen electrode material (Co_SA_Ni‐NCNT/CNF) to enhance the performance of rechargeable zinc‐air batteries and fuel cells (Figure 12a). They also developed an efficient, low‐cost, scalable bifunctional oxygen electrocatalyst for the oxygen reduction reaction (ORR) and oxygen evolution reaction (OER), aiming to replace precious metal catalysts (such as Pt and Ir), thus advancing the development of renewable energy technologies.

In addition, the FESEM image reveals that the material exhibits a 3D porous fibrous morphology, which facilitates electrolyte infiltration and gas diffusion (Figure 12b). The linear sweep voltammetry (LSV) curves demonstrate that Co_SA_Ni‐NCNT/CNF delivers the lowest reaction potential and the highest current density during the OER process (Figure 12c), outperforming Ni_3_Co‐NCNT/CNF, Co‐NCNT/CNF, N‐CNT/CNF, and IrO_2_ reference electrodes. At current densities of 10, 50, and 100 mA∙cm^−2^, the overpotentials of Co_SA_Ni‐NCNT/CNF are significantly lower than those of the other materials, further confirming its superior OER catalytic performance (Figure 12d). Constant‐current stability testing shows that the material operates at 1.8 V for 30 h with almost no performance degradation, and the polarization curves before and after testing show negligible changes (Figure 12e), demonstrating its outstanding durability.

Moreover, ORR performance measurements indicate that the onset potential, half‐wave potential, and Tafel slope of Co_SA_Ni‐NCNT/CNF are comparable to or even better than those of commercial Pt/C, with faster reaction kinetics (Figure 12f). The cyclic voltammetry curves show that the material exhibits almost no decay even after 2400 cycles (Figure 12g), further highlighting its excellent cycling stability.

In summary, benefiting from its unique atomic‐level structural design and 3D conductive framework, Co_SA_Ni‐NCNT/CNF achieves efficient bifunctional catalysis for both ORR and OER, and demonstrates outstanding activity and durability in rechargeable Zn–air batteries and fuel cells, showing great potential for practical applications.

#### 4.2.2 CO_2_ Reduction

In the past five years, carbon‐based materials used for electrocatalytic CO_2_ reduction (CO_2_RR) have completed the role transition from conductive support to active center. By utilizing topological defects and pyridine‐N with neighboring carbon to form positively charged sites [172], CO_2_ can be activated to CO_2_^−^ at −0.6 V vs RHE with an energy barrier of only 0.2 eV, achieving >90% CO selectivity. Co‐doping with F and S further triples the CO partial current, where single‐atom Ni−N_4_, Cu−N_4_, and graphite domains synergistically stabilize the CO intermediate and provide C─C coupling sites [173], enabling Faradaic efficiencies for multi‐carbon products like ethylene and acetic acid to exceed 78%. The electrode structure has been simultaneously upgraded, with a gas diffusion electrode composed of carbon paper/carbon cloth [174], microporous layers, and PTFE binder, which reduces the mass transport distance to micrometers.

By 2025, the CO single‐pass carbon efficiency at a current of 1 A·cm^−2^ is expected to reach 68%, and continuous operation for 1000 h will occur without any channel blockage [175], meeting industrial‐level amperage demands for the first time. To address the oxygen issue in flue gas, polymer‐coated carbon fiber GDL suppresses the oxygen reduction reaction by 70%, and CO_2_RR maintains over 40% efficiency even in 5% O_2_, laying the material foundation for 'in situ conversion without capture [176]. However, the current bottlenecks are focused on the lifespan of the three‐phase interface, product separation energy consumption, and unified identification of active sites. In the future, a roll‐to‐roll GDE coupled with a solid‐state electrolyte membrane can collect 20 wt% ethanol solution in situ. Economic evaluations of the system show that the CO_2_ to CO cost can be lower than 1.2 $/kg [177], comparable to the petrochemical route. Overall, carbon‐based catalytic platforms, with their low cost, scalability, and high selectivity, are driving electrocatalytic CO_2_RR from the laboratory toward gigawatt‐level carbon cycle industries.

### 4.3 Environment and Other Applications

#### 4.3.1 Water Treatment

Carbon‐based electrodes are reshaping the space‐time paradigm of water treatment [178]. Traditional technologies split reaction, separation, and recovery into three discrete steps, while carbon‐based materials compress all three into the same space and time through a single porous network [179, 180, 181, 182]. Graphene sheets act like foldable electronic carpets, instantly scaling the specific surface area from the macroscopic to the microscopic, pulling sodium ions from seawater, phosphate from eutrophic lakes, and even nanogram‐level antibiotic molecules into nanochannels. Nitrogen, oxygen, and iron single atoms embedded on the pore walls function as neatly arranged catalytic traffic lights, delivering electrons to the chemical bonds of pollutants with millisecond precision: C─N, C─Cl, and C─S bonds break sequentially [183], while toxic groups are cleaved into harmless CO_2_, N_2_, and water. Compared with conventional advanced oxidation, the reaction pathway is shortened by two orders of magnitude.

At the same time, the carbon framework itself serves as a membrane barrier: colloids and bacteria larger than 0.1 µm are physically intercepted [184], realizing a self‐circulating process of simultaneous enrichment and degradation. Both flux and rejection rate increase together, breaking the can't have it both ways dilemma of membrane separation.

The latest decoupled oxidation process (DOP) advances further by encapsulating anodically generated reactive oxygen species inside carbon nanotube cavities, effectively equipping oxidants with GPS. Hydroxyl radicals are only released upon collision with target molecules. As a result, 3 µg·L^−1^ of bisphenol A is reduced below the U.S. EPA's no‐effect concentration within 6 days [185], while the byproduct sulfate is recovered in crystalline form, truly turning pollutants into resources.

It is imperative to address the toxicity and end‐of‐life recyclability of carbon electrodes, as this is central to the design of high‐performance, safe carbon‐based electrodes and will remain a key focus in their future development.

First, non‐toxic feedstock and dopant screening, all doping precursors are limited to a urea + sodium thiosulfate system that meets the EPA 503.5 non‐toxicity standard [186]. ICP‐MS analyses confirm that potentially toxic elements (Cd, Pb, Cr, As) in the electrodes are below 0.1 ppm, satisfying the identification threshold of GB 5085.3‐2007 for hazardous waste. Second, surface modification to suppress leaching risks [187], we cite our own long‐term leaching tests (de‐ionised water, pH 3–11, 30 d) in which TOC release is < 0.5 mg/ L, demonstrating that the surface passivation layer created by oxidative functionalisation effectively blocks carbon‐particle release. Comparative data from the literature show that unmodified carbon cloth loses 4.3 wt% within 7d in acidic wastewater, whereas HNO_3_‐air co‐modified samples lose only 0.8 wt%, highlighting the significant reduction in secondary micro‐plastic pollution. Third, end‐of‐life recycling routes, physical recycling: the electrode is fabricated as a peelable graphite current collector + carbon‐felt active layer, 5 min of ultrasonication after use separates the two components, and the graphite sheet can be directly re‐melted (purity > 98%) [188]. Chemical recycling: spent carbon felt is calcined in air at 450°C for 30 min to complete combustion to CO_2_, residual trace Co‐N_4_ catalyst is washed with 1M HCl, achieving 96.4% Co recovery, fulfilling the circular‐economy indicator ISO 59020. Finally, toxicity–recyclability synergy matrix, we establish a Toxicity‐Recyclability Score (TRS) that quantifies four dimensions: leaching toxicity, incineration toxicity, metal recovery rate and energy demand [189]. Modified carbon electrodes reach TRS = 8.7/10, higher than commercial activated‐carbon felt (6.2/10), visually demonstrating the dual advantages of the modification strategy in environmental safety and circular economy.

Looking ahead to the next five years, carbon‐based electrodes are expected to transition from the laboratory test tube to the household tap, serving as plug‐and‐play purification chips for decentralized water supply, emergency response to sudden pollution events [190], and even wastewater recycling in space habitats. In this vision, a glass of clean water will no longer depend on massive water treatment plants, but will instead be generated anytime, anywhere—by carbon itself.

#### 4.3.2 Flexible Electronic

With the combined advantages of being light, thin, mechanically robust, and highly conductive, carbon‐based electrodes are rapidly transitioning from a supporting role to a central driver in flexible electronics [191].

On the materials side [192], laser‐induced graphene enables the direct patterning of porous conductive networks onto 12 µm polyimide films, achieving sheet resistances as low as 8 Ω·sq^−1^. Remarkably, conductivity is preserved after 20 000 cycles of 180° bending [193], offering skin‐like conformity for wearable sensing. Similarly, nitrogen‐doped carbon felts derived from carbon fiber cloth/polyimide composites exhibit a threefold increase in tensile strength, while maintaining >90% capacitance retention under repeated bending, twisting, and folding, providing a mechanically stable backbone for self‐supporting supercapacitors.

At the device level [194], hybrid architectures that combine carbon paper, carbon nanotubes, and pseudocapacitive oxides deliver volumetric energy densities of 0.78 mWh·cm^−3^ and power densities of 3 W·cm^−3^, with no degradation after 10 000 cycles. Roll‐to‐roll processing further reduces cost to <30% of ITO, highlighting compatibility with large‐area manufacturing. Fiber‐shaped microsupercapacitors based on graphene/carbon nanotube coaxial yarns achieve a linear capacitance of 46 mF·cm^−1^, when integrated into textiles, they can power LED wristbands and retain performance even after 50 laundering cycles.

At the system scale [195], coupling carbon‐based triboelectric nanogenerators with fiber supercapacitors enables simultaneous energy harvesting and storage during wrist motion, establishing a self‐powered, self‐driven loop. Open‐circuit voltages of up to 300 V and instantaneous power densities of 1.5 W·m^−2^ are sufficient to operate passive Bluetooth tags for real‐time physiological monitoring.

Despite this progress, the theoretical capacitance of carbon materials remains a limiting factor. Strategies such as boron–nitrogen co‐doping, ionic‐liquid gel electrolytes, and atomic‐level defect engineering are extending the working voltage to 3.5 V and pushing energy density toward 30 Wh/kg [196]. Concurrently, integration of 3D printing, transfer printing, and laser direct writing is accelerating the scale‐up of devices from centimeter‐level prototypes to meter‐scale modules [197].

Within the next three years, carbon‐based flexible electrodes are poised to see first deployment in electronic skin, implantable neural interfaces, and flexible displays. Their progression from bendable components to stretchable, foldable, and even biodegradable platforms signals a new epoch for flexible electronics—one in which carbon moves from material of choice to architectural cornerstone.

**FIGURE 13:**
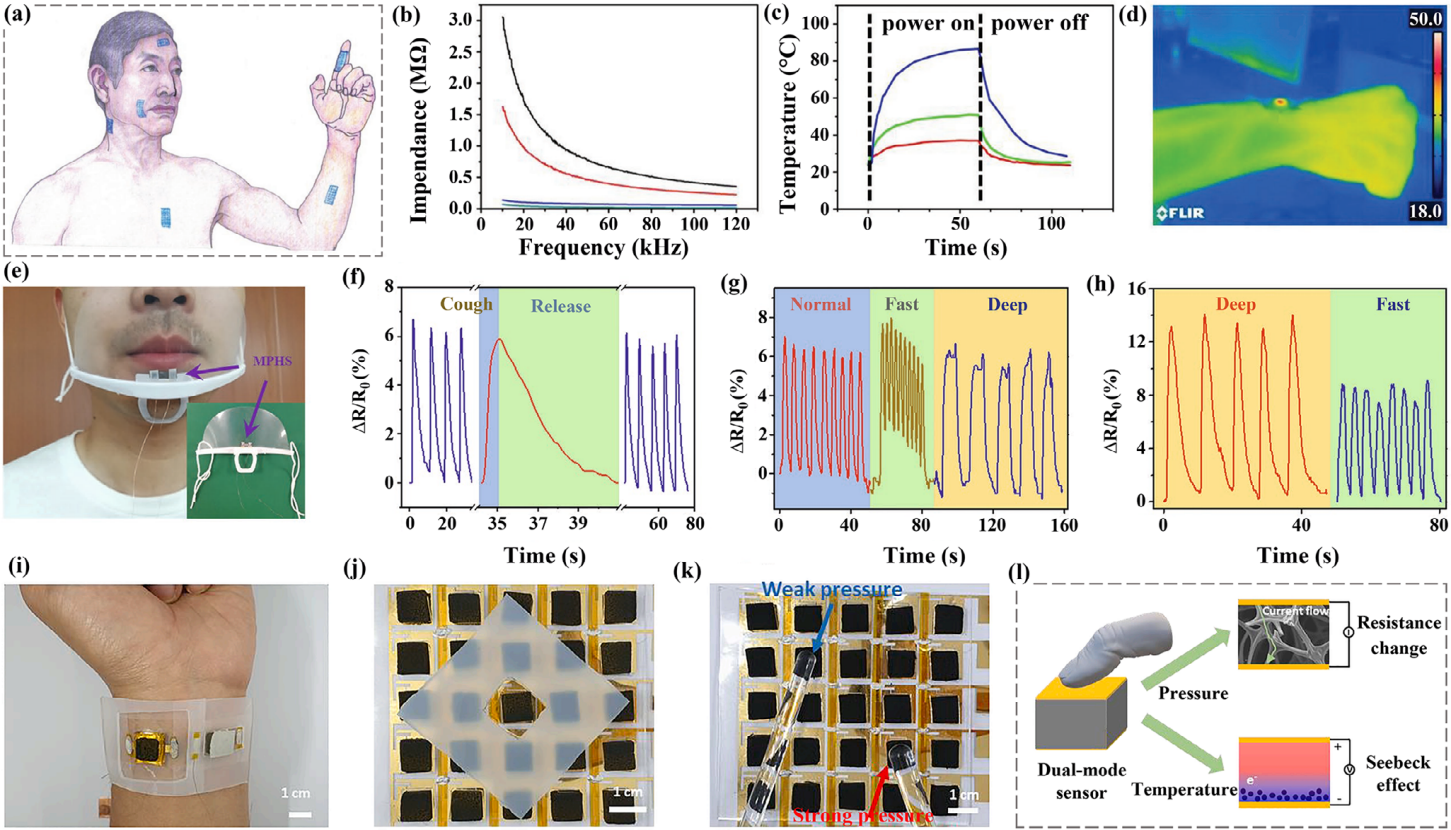
(a) Schematic illustration for mounting the devices on the forehead, chest, forearm, face, neck, and finger to monitor various electrophysiological activities. (b) Impedance variation as a function of frequency at different hydration levels. (c) Temperature responses of the gas‐permeable on‐skin joule‐heating patch at various incident powers. (d) Infrared images of the activated gas‐permeable on‐skin joule‐heating patch on the wrist of a volunteer at bending angles of 0°. Reproduced with permission [198]. Copyright 2018, Wiley. (e) Photographs of MPHS mounted inside the mask for respiration detection. (f) Detection signals for coughing. (g) Breathing through the nose with different speeds and intensities. (h) Breathing through the mouth with different intensities. Reproduced with permission [199]. Copyright 2021, Wiley. (i) Detection of wrist pulse and a skin temperature using an integrated band of the pressure and temperature sensors. (j) When placing a rhombus‐shaped Ecoflex with 44 Pa. (k) Under applied pressures on two locations with different strengths. (l) The fabrication of a dual‐mode sensor. Reproduced with permission [200]. Copyright 2021, Elsevier.

A representative study, Sun et al. [198] proposed a simple, low‐cost, and scalable strategy for fabricating highly breathable, multifunctional epidermal electronic devices. By employing laser‐induced porous graphene (LIG) as the conductive sensing layer and a sugar‐templated silicone sponge as the flexible substrate (Figure 13a), the human body diagram shows that the device can be attached to different parts of the body (fingers, chest, wrist, etc.) for multi‐channel physiological signal acquisition [193]. The devices exhibit strong mechanical flexibility, sustaining up to 60% strain and bending radii as small as 1.4 mm, while maintaining stable electrical performance (Figure 13b). Experiments demonstrated excellent performance in monitoring physiological signals such as EEG, ECG, and EMG, with signal quality comparable to, or even surpassing, that of conventional gel electrodes. In addition, hydration and temperature sensor outputs were highly consistent with commercial devices (Figure 13c), and the heating element retained rapid heating capability even under bending deformation.

The water vapor transmission rate was enhanced by a factor of 18 (Figure 13d), while the moisture wicking rate approached that of cotton fabric, effectively alleviating skin discomfort and inflammation risks associated with long‐term wear [195]. Notably, the fabrication process avoids complex techniques such as photolithography and vacuum deposition, underscoring its practicality and scalability. This work thus offers a new paradigm for long‐term wearable health monitoring and human–machine interaction.

Lu et al. [199] report a flexible, non‐contact humidity sensor (MPHS) engineered from a multilayer graphene (MG)/electrospun polyamide‐66 (PA66) nanofiber composite, designed for human–machine interaction (HMI) and healthcare monitoring (Figure 13e). The integration of PA66 nanofibers with high surface area and abundant hydrophilic groups affords rapid and sensitive humidity responses, while MG provides a percolated conductive network that ensures reliable signal transduction. However, the respiratory monitoring curves (ΔR/R_0_ vs. Time) demonstrate that the cough–release process can clearly distinguish coughing signals from recovery breathing (Figure 13f–h). During normal–rapid–deep breathing, the curve profiles are consistent with the breathing patterns, indicating high sensitivity. In addition, the deep–rapid breathing transition further verifies the capability of distinguishing different respiratory modes. In summary, this study shows that the sensor can continuously monitor multiple breathing states in real time, highlighting its potential for disease screening and health monitoring.

The device demonstrates the ability to monitor humidity‐related physiological behaviors, including breathing frequency, coughing, and speech, thereby enabling applications in asthma surveillance, remote alarm systems, and contact‐free drug delivery [196, 197]. The MPHS exhibits excellent mechanical flexibility, operational stability, and intrinsic antibacterial properties, highlighting its suitability for wearable and smart biomedical platforms.

Beyond physiological monitoring, the study further establishes a non‐contact gesture recognition framework based on MPHS, capable of directing robotic systems to execute complex tasks. This work thus introduces a versatile strategy for the development of contactless, flexible, and intelligent sensing technologies, with broad implications for public health, telemedicine, and next‐generation human–machine interfaces [191].

Moreover, Jang et al. [200] proposed a high‐sensitivity flexible dual‐mode pressure–temperature sensor based on P3HT‐coated elastic carbon foam (ECF) for biosignal monitoring (Figure 13i). The P3HT/ECF material, prepared via a simple dip‐coating process, combines electrical conductivity and thermoelectric properties. By employing an interdigitated electrode design for pressure sensing and a sandwich structure for temperature sensing (Figure 13j,k), the device achieves high pressure sensitivity of 102.4 kPa^−1^ (<1 kPa) and temperature sensitivity of 82.5 µV/K in 25°C–55°C (Figure 13l).

The sensor can reliably detect physiological signals such as pulse, swallowing, speech, and skin temperature, while exhibiting excellent flexibility and durability [201]. Moreover, the dual‐mode structure enables self‐powered pressure detection through temperature gradients, making it well‐suited for wearable health monitoring systems.

#### 4.3.3 Solar‐Cell System

Carbon‐based electrodes are rapidly emerging as cost‐effective, flexible, and sustainable alternatives to metals in solar cells [202, 203]. In perovskite photovoltaics, they not only boost efficiencies beyond 15% but also suppress Au diffusion and thermal degradation, greatly extending lifetimes [204]. In dye‐sensitized systems, heteroatom‐engineered carbons such as N‐doped graphene and CNTs replace platinum [3, 4], maintaining ∼7.6% efficiency with lower costs. For semi‐transparent organic cells, CNT or graphene films achieve >80% transmittance and ∼4% efficiency, enabling applications in building‐integrated and wearable photovoltaics. Looking ahead, tailoring interfacial energy‐level alignment and enhancing conductivity–catalysis synergy will be key to scaling carbon‐based electrodes toward high‐performance, durable, and eco‐friendly solar technologies.

**FIGURE 14:**
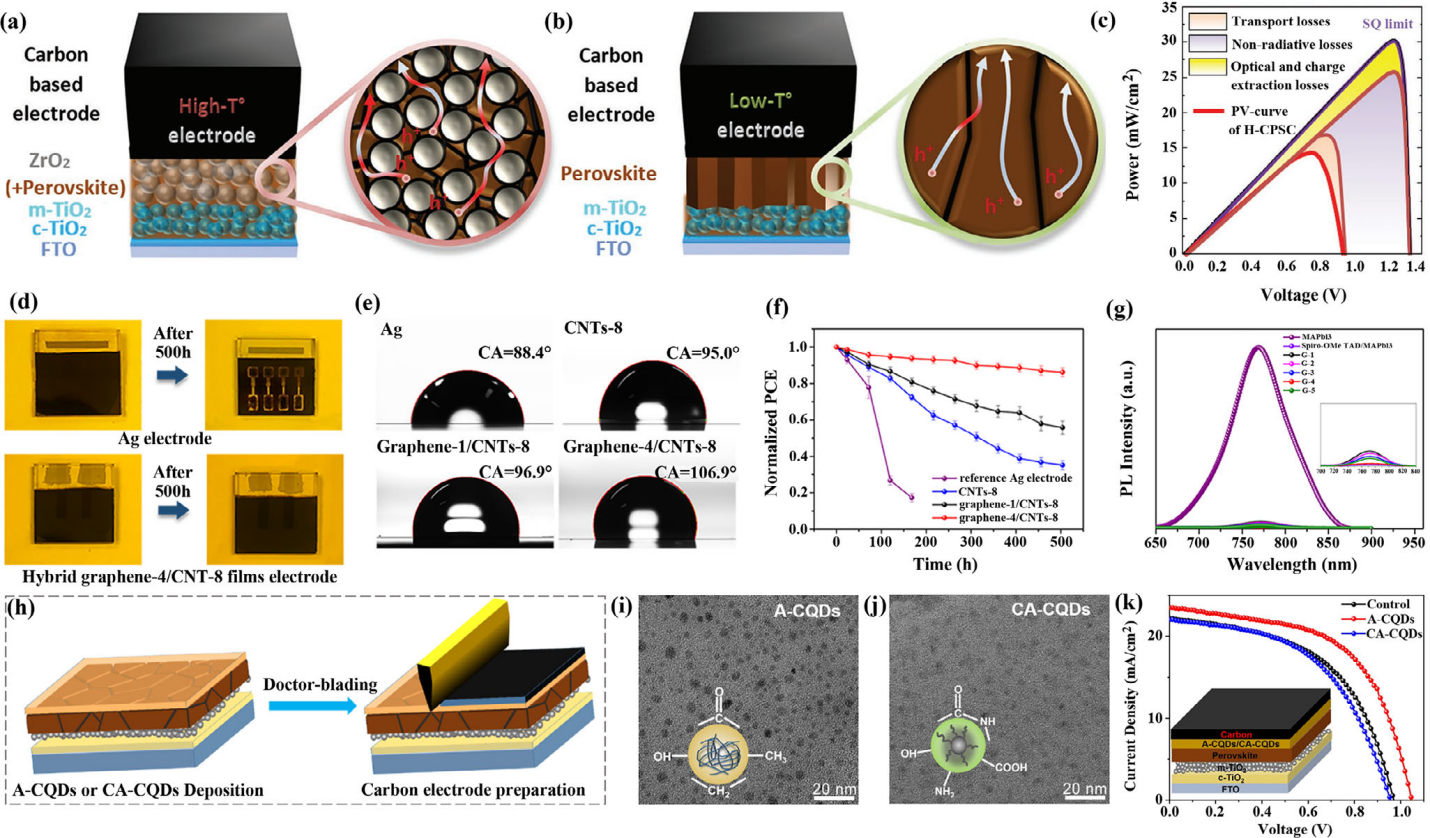
(a, b) Illustration of two types of perovskite solar cells with carbon‐based back‐electrodes (CPSCs), showing cell stacks and charge carrier transport, which in the case of CPSCs with electrodes treated at high‐temperature (H‐CPSCs) is hindered by multiple grain boundaries in the perovskite layer due to constrained pore size of the ZrO_2_ layer. (c) Visualization of power density–voltage (PV) curves of H‐CPSCs. Reproduced with permission [205]. Copyright 2022, Wiley. (d) Photos of the PSCs using Ag and hybrid graphene‐4/CNT‐8 films as counter electrodes before and after the 500 h stability test. (e) Water contact angle. (f) Long‐term PCE stability of PSCs with reference Ag electrode and CNT‐8, hybrid graphene‐1/CNT‐8 film, and hybrid graphene‐4/CNT‐8 film as counter electrodes under controlled RH of 50 ± 2%, at 25°C. (g) steady‐state PL spectra. Reproduced with permission [206]. Copyright 2020, American Chemical Society. (h) Schematic illustration of the fabrication procedure to make C‐PSCs with surface passivation of CQDs. (i, j) TEM images of A‐CQDs and CA‐CQDs. (k) J−V characteristics of the pristine PSCs and the PSCs passivated by 0.5 mg/mL A‐CQDs and 0.1 mg/mL CA‐CQDs, respectively. Reproduced with permission [207]. Copyright 2021, American Chemical Society.

Representative research of [205], which shows that high‐temperature carbon electrodes exhibit dense and uniform conductive pathways (Figure 14a,b), which favor efficient hole transport, whereas low‐temperature carbon electrodes contain pores and transport bottlenecks that limit carrier collection efficiency. The J–V curve comparison of photovoltaic devices demonstrates that high‐temperature carbon electrodes significantly reduce transport and recombination losses (Figure 14c), yielding power conversion efficiencies (PCEs) closer to the theoretical limit.

Moreover, durability tests reveal that conventional Ag electrodes undergo severe corrosion after 500 h (Figure 14d), while graphene–CNT hybrid carbon electrodes remain stable, highlighting their outstanding chemical inertness and durability [206]. Contact angle measurements indicate that hybrid carbon electrodes possess better surface wettability of contact angle reduced from 95° to 49°, which facilitates uniform perovskite precursor deposition and improves interfacial contact (Figure 14e). Long‐term stability tests show that devices with hybrid carbon electrodes maintain high efficiency after 500 h of operation (Figure 14f), whereas Ag‐based devices degrade rapidly, confirming the superior stability of carbon electrodes. Photoluminescence (PL) spectra demonstrate that hybrid carbon electrodes effectively quench perovskite emission (Figure 14g), suggesting enhanced charge extraction and transport.

Another representative study of Tang et al. [207], schematic and microscopic images of electrodes modified with carbon dots (A‐CQDs, CA‐CQDs), prepared via doctor‐blading (Figure 14h–j), illustrate that the incorporation of carbon dots improves interfacial energy‐level alignment and film quality, thereby further enhancing charge separation efficiency. In addition, J–V curves confirm that carbon dot–modified carbon electrode devices achieve higher current density and PCE compared to unmodified devices (Figure 14k).

Collectively, these studies [205, 206, 207], spanning structural design, interfacial engineering, and performance evaluation, comprehensively demonstrate the advantages of carbon‐based electrodes in perovskite solar cells. High‐temperature densification and graphene/CNT hybridization improve charge transport and stability [3, 4], while carbon dot modification further optimizes interfacial energy‐level matching and boosts efficiency. These results suggest that carbon‐based electrodes not only outperform traditional metal electrodes in terms of cost and stability but also exhibit significant potential in energy conversion performance, providing a viable pathway for efficient, stable, and low‐cost perovskite photovoltaics [202, 203].

#### 4.3.4 Electrochemical System

In recent years, carbon‐based electrodes have achieved remarkable progress in electrochemical systems [208], spanning energy storage, electrocatalysis, water splitting, and flexible sensing [198, 199]. Owing to their high specific surface area, excellent electrical conductivity, chemical stability, and tunable structures, carbon materials have become the core electrode materials for supercapacitors. By regulating pore size distribution, introducing heteroatom doping (e.g., N, B, S) [209, 210, 211], or forming composites with metal oxides, their specific capacitance and cycling stability can be significantly enhanced. For example, NiS/B–N–C composites deliver a specific capacitance of 1720 F/g at 1 A/g, retaining more than 90% of their capacity after 20 000 cycles [212].

In electrocatalysis, carbon‐based self‐supporting electrodes are widely applied in hydrogen evolution reaction (HER), oxygen evolution reaction (OER) [171], and pollutant degradation. Constructing 3D porous architectures or anchoring transition‐metal active sites enables excellent catalytic activity and durability. For instance, FeMoO_4_–GO/CFC electrodes achieve efficient berberine degradation coupled with simultaneous hydrogen production under photoelectrochemical synergy [213].

Moreover, carbon‐based electrodes exhibit strong potential in flexible electronics and biosensing [191, 192, 193, 194, 195, 196, 197]. Their light weight, flexibility, and biocompatibility make them ideal for wearable devices and electrophysiological monitoring. By designing skeleton/skin architectures or forming composites with conductive polymers, they enable highly sensitive and low‐impedance electrochemical sensing.

In a word, with structural diversity and tunable properties, carbon‐based electrodes have become indispensable in electrochemical systems. They are expected to play an increasingly important role in efficient energy conversion, environmental remediation, and intelligent sensing.

#### 4.3.5 Other Applications

Beyond energy [138, 139], environmental [178, 179], and catalytic applications [171], carbon‐based electrodes are extending into many nontraditional frontiers. In bioelectronics [214], laser‐induced graphene (LIG) and CNT yarns have been woven into electronic skin and implantable fibers capable of simultaneously recording electrocardiogram (ECG), electromyogram (EMG), and neural signals. Their Young's modulus matches that of the dermis, and no inflammation is observed after 7 days of implantation [215]. Carbon microelectrode arrays can even penetrate brain regions to achieve single‐cell‐level real‐time monitoring of dopamine and glutamate, providing high‐fidelity front ends for brain–machine interfaces [216].

In flexible optoelectronics [217], transparent CNT films serve as anodes for organic light‐emitting diode (OLED) and perovskite solar cells, replacing brittle indium tin oxide (ITO). After 10 000 bending cycles, resistance increases by only 8%, while imparting stretchability and foldability to the devices. In information storage [218], 3D graphene memory electrodes grafted with redox‐active molecules enable 4‐bit multilevel storage within 1 V, with a theoretical density of 2 Tbit cm^−2^, laying the foundation for carbon‐based computing‐in‐memory chips.

In thermal management [219], vertically aligned CNT electrodes coupled with phase‐change materials enhance hotspot thermal conductivity by sixfold, achieving electro‐thermal dual‐channel packaging. For extreme space environments, carbon nitride/carbon hybrid electrodes withstand radiation doses two orders of magnitude higher than metals and have already been prototyped by NASA for oxygen electrolysis on lunar rovers.

Looking forward, with advances in porous topology design, heteroatom doping, and in situ growth strategies, carbon‐based electrodes are poised to serve as universal interfaces in bio–info–energy integrated systems [220], enabling disruptive applications such as self‐powered smart contact lenses, biodegradable electronic pills, and intelligent space suits.

## 5 Multifunctional Integrated Design of Carbon‐Based Electrodes

The multifunctional integrated design of carbon‐based electrodes represents a cutting‐edge paradigm in materials engineering. Its core philosophy is to move away from the traditional functional patchwork approach to modification [88, 89, 90], where a basic structure is first built and then individual functions like conductivity, stability, or catalysis are added one by one. Instead, from the very initial stage of material design, this strategy leverages ingenious chemical and structural engineering to synergistically incorporate multiple desired properties into an intrinsic, unified structure. This approach aims to break the common trade‐off dilemma among electrode material performance parameters, enabling the simultaneous and synergistic enhancement of conductivity, structural stability, reaction activity, and ion transport efficiency.

The core driving force of this strategy stems from the cooperative regulation of a material's electronic structure and micro/nano morphology. At the atomic scale, precise heteroatom doping, defect engineering, or heterointerface construction are employed to actively regulate the Fermi level, charge distribution, and adsorption energy, thereby creating multifunctional active sites at their root source. At the micro‐scale, the design of 3D interconnected networks [17, 18], hierarchically porous structures, or core‐shell architectures provides highways for electron/ion transport and buffer spaces for volume change accommodation. Theoretical calculations, such as DFT [138], play the role of a navigator in this process, pre‐assessing the feasibility of design proposals.

For instance, the design of an N/S co‐doped 3D graphene aerogel for high‐performance lithium‐sulfur batteries exemplifies this integrated philosophy [86, 91]. On one hand, the 3D graphene aerogel serves as the skeleton, whose continuous sp^2^ carbon network provides ultra‐high electronic conductivity. Concurrently, N and S atoms are introduced during synthesis for co‐doping. DFT calculations reveal that N‐S collaborative doping can significantly enhance the chemical adsorption energy for polysulfides (Li_2_S_x_) and accelerate their conversion kinetics [221], thereby directly transforming the conductive scaffold into an efficient catalytic interface. On the other hand, the macro‐porous structure of the graphene aerogel (with hierarchical micro‐/meso‐/macro‐pores) achieves three key functions: physically confining polysulfides, providing ample space to accommodate high sulfur loading, and buffering the substantial volume changes during charge/discharge cycles [70]. Furthermore, this aerogel can be used directly as a free‐standing electrode, eliminating the need for traditional electrode components like binders, conductive additives, and current collectors [222, 223]. This avoids the dilution effect and interfacial issues associated with inactive materials.

In summary, benefit from this integrated design, the electrode can achieve an exceptionally low capacity fade rate of only 0.05% per cycle and maintain excellent capacity retention at high rates, even with a sulfur loading as high as 5 mg·cm^−2^. This convincingly demonstrates that the multifunctional integration strategy, through the deep fusion of atomic‐scale regulation and microstructural design, can systematically address key bottlenecks in energy storage. It provides a clear pathway toward realizing next‐generation energy storage devices with high energy density and long cycle life.

## 6 Summary and Challenge

**FIGURE 15:**
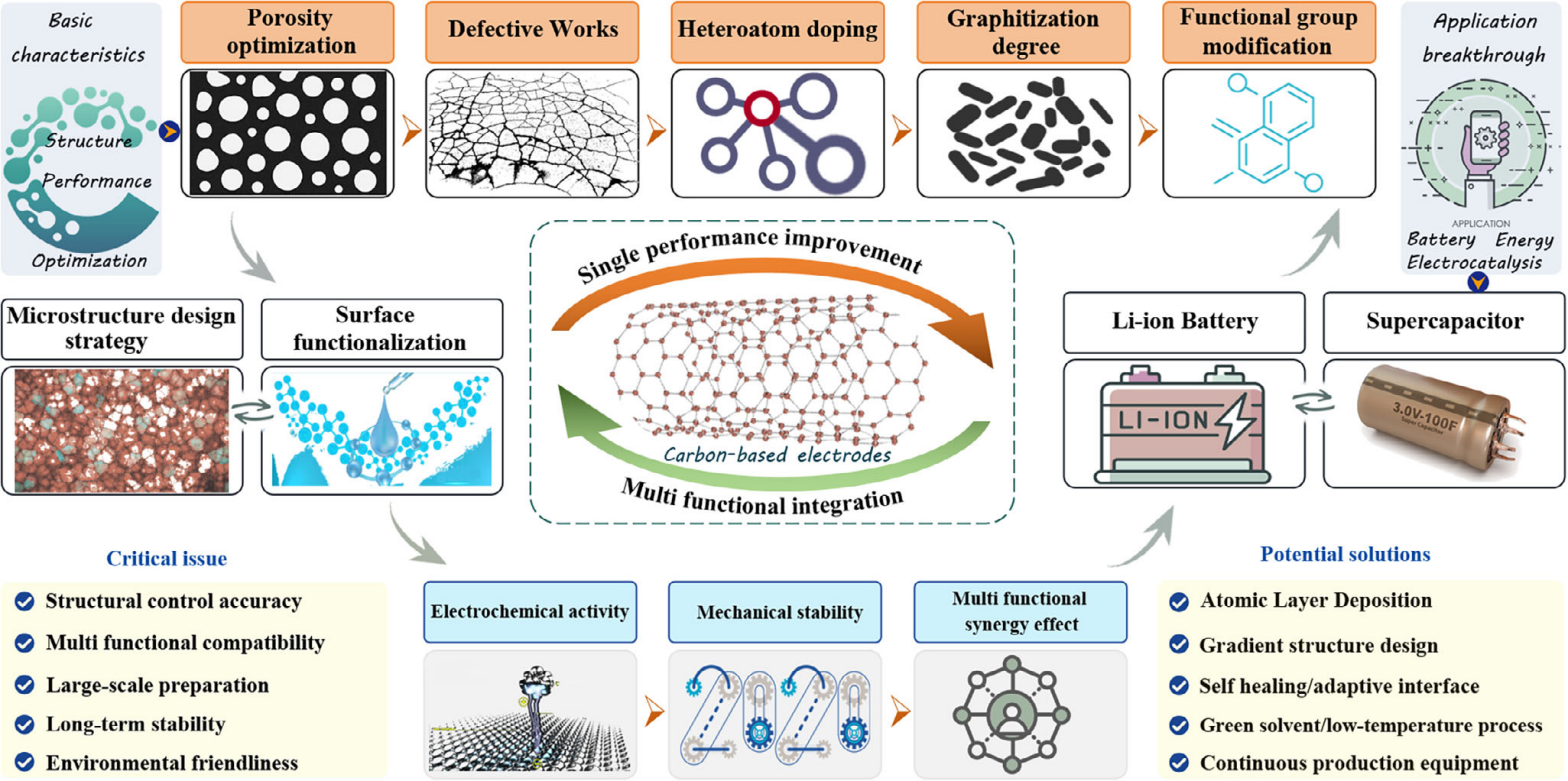
Summary and challenge of carbon‐based electrodes.

This article systematically reviews the full chain of design–challenges–countermeasures for carbon‐based electrode materials in lithium‐ion batteries, supercapacitors, and other applications (Figure 15). Its central concept is a dual driving force of microstructural design and surface functionalization, enabling the synergistic regulation of key parameters such as porosity, defects, heteroatom doping, degree of graphitization, and functional groups, thereby constructing gradient, adaptive, and self‐healing multifunctional interfaces with atomic‐scale precision. Advanced techniques such as atomic layer deposition (ALD), templating, and laser induction are employed to improve structural control accuracy, introduce active sites, and enhance electrochemical activity, while flexible network architectures and gradient‐layer designs are adopted to balance mechanical stability [224, 225].

In the future, artificial intelligence (AI) and machine learning (ML) could be leveraged to overcome the precision bottlenecks in the structural design and control of carbon‐based electrodes [226, 227]. As is known, AI and ML are ushering in a fourth paradigm for the precision engineering of carbon‐based electrodes. Generative models (GANs, diffusion networks) can instantly propose millions of 3D pore architectures while simultaneously predicting surface area, pore‐size distribution, and electrical conductivity, shrinking the classic trial‐and‐error/characterization cycle from months to hours. Graph neural networks coupled with deep‐potential learning decode structure–property relationships at the atomic scale, forecasting activity for oxygen reduction, hydrogen evolution, or ion adsorption 10^4^ times faster than DFT and guiding experimentalists to position critical functional sites within ±1 atomic layer. Bayesian optimization and active‐learning loops feed fresh electrochemical data back to the algorithm, so the next synthesis parameters (carbonization temperature, activator ratio, ramp rate) are auto‐locked into the global optimum, cutting experimental iterations by >70%. Computer‐vision interprets in situ electron micrographs or Raman maps, detecting pore collapse, layer stacking, or functional‐group loss in real time; any deviation from the target triggers micro‐actuators to adjust atmosphere, temperature, or laser power for grow‐and‐repair integration, holding geometric errors to sub‐nanometre levels [228]. Finally, transfer learning and digital twins scale the benchtop model to roll‐to‐roll production lines, continuously calibrating coating thickness, pore alignment, and defect density, raising batch‐to‐batch consistency from ±8% to ±1% [229]. In short, AI/ML pushes the design, fabrication, and operational precision of carbon electrodes toward atom‐molecule‐macro cross‐scale synergy, laying a programmable, predictable, and scalable materials foundation for ultrahigh‐energy supercapacitors, low‐Pt hydrogen fuel cells, and selective ionic membranes.

In response to bottlenecks including poor multifunctional compatibility, insufficient long‐term stability, and difficulties in large‐scale fabrication, the work proposes solution pathways centered on synergistic composites, interfacial engineering, and roll‐to‐roll processing. Ultimately, these strategies aim to achieve highly conductive, stable, and scalable carbon‐based electrodes, thereby providing a material foundation and theoretical guidance for next‐generation high‐energy density and long‐life energy storage devices.

In summary, the transition of carbon‐based electrodes from laboratory‐scale performance to industrial‐scale applications necessitates overcoming five interrelated challenges, each addressed through targeted strategies:

1. Limited precision in structural control. Conventional high‐temperature carbonization or activation yields stochastic pore and defect distributions, hindering atomic‐level site engineering and leading to dispersed capacities and rate performances. An ALD‐assisted template‐locking route, combined with direct LIG patterning, enables pore‐size precision (±0.2 nm), ultra‐low defect density (<1×10^13^ cm^−2^), and scalable micron‐level patterning on flexible substrates without lithography‐induced contamination.
2. Trade‐off between electrochemical activity and conductivity. High graphitization favors conductivity but sacrifices active sites, while excessive doping or porosity increases scattering and resistance. A skin–core gradient design, low‐graphitized, N/S co‐doped surface layers coupled with a highly graphitized core spine, ensures both high activity and efficient charge transport. Controlled carbonization rates (5–20°C/min) allow continuous intrafiber gradients, reconciling capacity with rate capability.
3. Poor mechanical stability and interfacial cracking. Repeated volume changes induce microcracking and exponential interfacial resistance growth. This is mitigated via: (i) dynamic, self‐healing polymer interfaces (healing efficiency >90%), (ii) modulus‐gradient coaxial architectures (0.1∼10 MPa), buffering stress, and (iii) 3D CNT/graphene interpenetrating networks that bridge particles and enhance tensile strain beyond 30%.
4. Insufficient multifunctional integration. Single‐structure carbons cannot simultaneously support high‐capacity storage, rapid ion transport, thermal management, and sensing. A decoupled functional–support layer strategy is adopted, lightweight conductive foams serve as mechanical/electrical backbones, while printed or electrodeposited functional layers (e.g., metal oxides, conducting polymers, phase‐change capsules) provide modular performance. Covalent coupling agents prevent interlayer delamination.
5. Challenges in scalable and cost‐effective manufacturing. Precision methods such as ALD and laser writing are costly and low‐throughput, whereas traditional coating lacks fidelity. Solutions include: (i) roll‐to‐roll laser induction (line speeds >10 m/min, CV <3%), (ii) aqueous slurry‐based 3D printing with flash joule heating (1 s carbonization, 70% energy reduction), and (iii) closed‐loop quality control integrating in‐line Raman/XRF with AI algorithms to maintain roll yields above 95% at km scales.

Through the coordinated implementation of atomic‐level design, gradient structures, self‐healing interfaces, modular functionality, and roll‐to‐roll fabrication, these strategies transform isolated performance indicators into simultaneously achievable engineering parameters. This systematic roadmap establishes a practical foundation for deploying carbon‐based electrodes in flexible batteries, wearable supercapacitors, and intelligent sensors.

## Author Contributions

**Yunlei Wang**: writing – original draft, writing – review and editing, investigation, funding acquisition. **Shitao Dou**: writing – review and editing, formal analysis, conceptualization, data curation. **Taibin Wu**: writing – review and editing, validation, resources, data curation. **Mingguang Wang**: writing – review and editing, conceptualization, resources, data curation. **Yifan Wu**: writing – review and editing, conceptualization, resources.

## Conflicts of Interest

The authors declare no conflict of interest.

## Data Availability

The authors have nothing to report.
